# Topological Transition in Aromatic and Quinonoid π-Conjugated Polymers Induced by Static Strain

**DOI:** 10.1021/jacs.4c10064

**Published:** 2024-09-13

**Authors:** Rameswar Bhattacharjee, Miklos Kertesz

**Affiliations:** Department of Chemistry, Georgetown University, Washington, District of Columbia 20057, United States

## Abstract

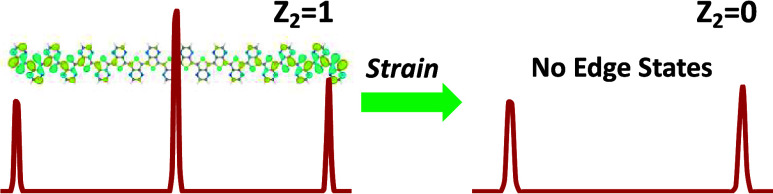

A topological quantum phase transition has been identified for the first time for 24 π-conjugated polymers as a function of external longitudinal strain due to a level crossing of the frontier orbitals at the topological phase transition. Topological phase is determined by the presence/absence of edge states. Out of the 24 polymers 15 are traditionally assigned an aromatic character, and 9 are traditionally assigned a quinonoid character. We find that all aromatic ones correspond to the trivial topological phase (Zak invariant, *Z*_2_ = 0), while all of the quinonoid ones to the nontrivial topological phase (*Z*_2_ = 1) replacing the intuitive characterization of aromatic/quinonoid with the physically well-defined Zak invariant. Unique topological phase transition as a function of tensile strain can be achieved for four of the quinonoid ones. Tensile strain in these cases leads to a reduction of the bandgap. We introduced a figure of merit for indicating the efficiency of achievable very small bandgap upon the application of external strain.

## 1 Introduction

**Figure 1:**
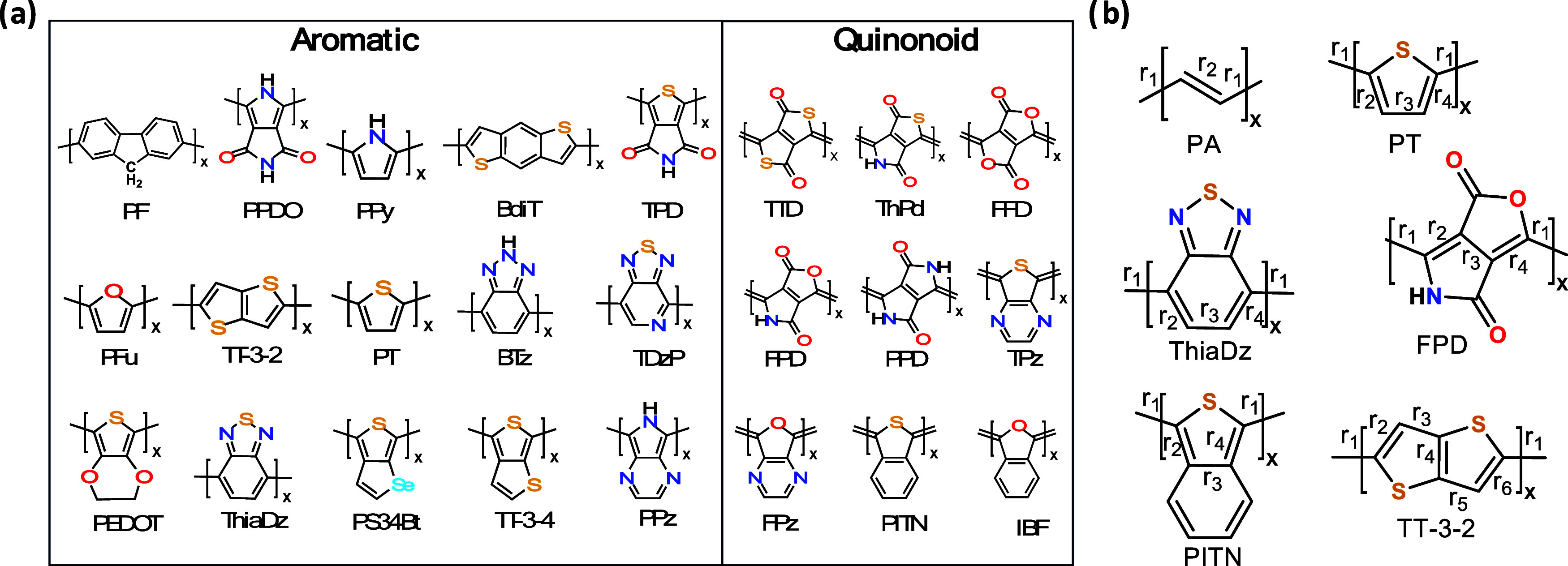
(a) Chemical repeat units of π-conjugated polymers discussed in this work. Left side lists aromatic ground state systems (A), the right side are quinonoid (Q) as defined in the text. (b) Carbon–carbon bonds for selected polymers. *r*_1_ is the length of the carbon–carbon bond connecting adjacent repeat units. The other *r_i_* bond lengths are used to define the bond length alternation (BLA) parameter in eqs 1 and 2. For chemical names, see Table S1.

Topological insulators are exciting materials because they offer unusual combinations of physical properties with potential applications in the class of one-dimensional (1D) materials as polymers.^1^ In the realm of π-conjugated polymers, such as those illustrated in Figure 1, they offer localized spins at the termini (edge states, or end states), extremely low bandgaps, and quasi-metallic properties without doping.^2−8^

**Figure 2:**
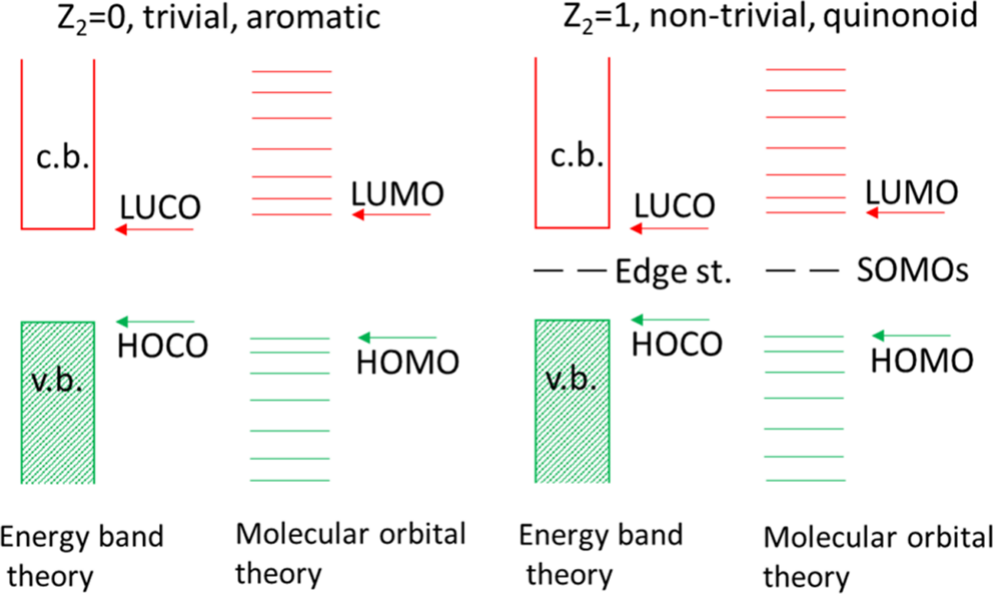
Schematic comparison of the terminologies used. HOCO: highest occupied crystal orbital, LUCO: lowest unoccupied crystal orbital, HOMO, LUMO are the respective molecular orbitals. SOMOs are the two singly occupied molecular orbitals corresponding to the to edge states in the solid-state terminology. Conduction and valence bands are indicated by c.b. and v.b., respectively.

Recent experiments on π-conjugated ethynylene bridged [n]acene polymers fabricated on Au(111) surfaces coupled with theoretical investigations concluded that these display a topological phase transition as a function of the linked acene size (*n*) between anthracene (*n* = 3) and pentacene (*n* = 5). Cirera et al. identified this transition as an aromatic to a quinonoid transformation resulting from the highest occupied molecular orbital–lowest unoccupied molecular orbital (HOMO–LUMO) (highest occupied vs lowest unoccupied) level crossing along the discrete chemical variation as the size of the molecular components along the *n* = 1, 3, 5, 7, 9 series is varied.^2^ They linked this transition to the concept of trivial (*Z*_2_ = 0) to nontrivial (*Z*_2_ = 1) topological phase transition, where *Z*_2_ is the Zak invariant.^9^ In a true transition, however, there should be a physical parameter that can be continuously varied and this work addresses that such a parameter can be the external strain. Key characteristics for these two alternatives are listed in Figure 2 by comparing the two commonly used terminologies, one referring to the solid state, the other to molecules.

**Figure 3:**
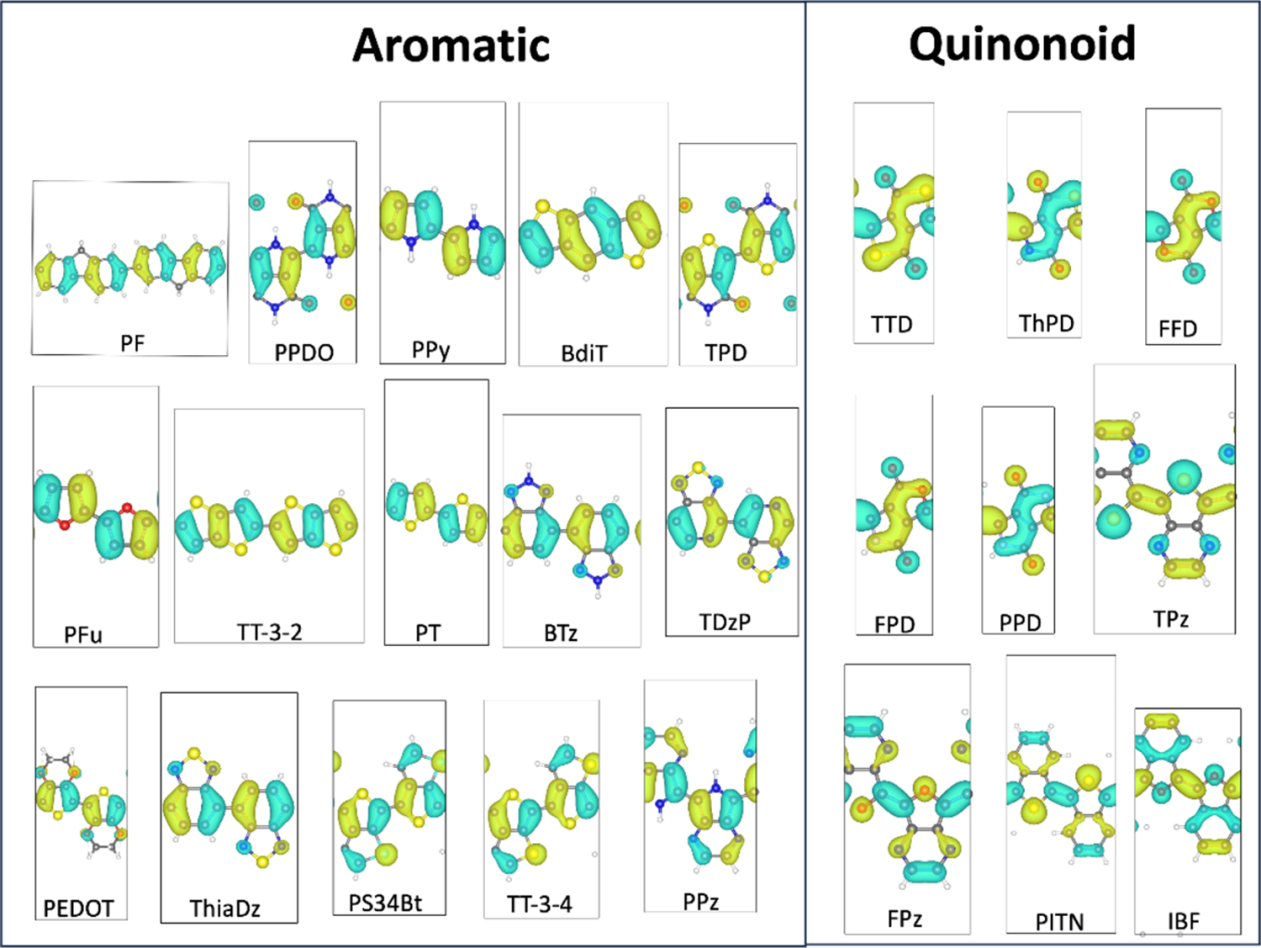
Top view of the HOMOs for the polymers investigated are shown. All refer to π-type orbitals, i.e., they all have a node in the plane of the system. All orbitals depicted refer to the *k* = 0 point in the 1D Brillouin zone.

Most π-conjugated polymers display aromatic units connected by formally single bonds, as in polythiophene, PT, and the other 14 listed on the left in Figure 1a. These will be denoted as “aromatic” (A). Wudl et al.^10^ discovered the first quinonoid (Q) π-conjugated polymer, polyisothianaphthene, PITN, in their quest for low bandgap polymers. In PITN and the other eight Q polymers listed in Figure 1a, the units are connected by formal double bonds with fundamentally different properties than the aromatic ones. A basic parameter that helps to distinguish these two categories is the interunit carbon–carbon bond distance,^11^*r*_1_, illustrated in Figure 1b. The definition of whether a polymer belongs to the A or Q category is complicated by the fact that due to their diverse structures, the character of only one carbon–carbon (CC) bond can be insufficient, and a more subtle definition is needed that relies on the phases of the HOMOs between the unit cells. For aromatic structures, the HOMOs on the adjacent carbon atoms linking the unit cells are out of phase, and for quinonoid structures they are in phase as illustrated in the orbital diagrams in Figure 3.

Certainly, reasonable VB structures of these systems can be legitimately drawn for both aromatic and quinonoid structures to represent their ground states. However, our full geometry optimization computations produced unique equilibrium ground state structures in all cases. The *r*_1_ values and gaps shown later on (Figure 6) provide limited categorization, but the orbital phases of the HOMO and LUMO at the r_1_ linking group make the categorization (A or Q) unique. This is then reinforced by the value of the topological Zak invariant.

For most of these polymers, a useful insight is available connecting their bond distance patterns through a bond length alternation parameter, BLA, to their bandgaps, *E*_g_.^12−14^ This relationship is summarized below. The level crossing as a function of BLA for polyacetylene, PA, is a manifestation of the Peierls distortion^15^
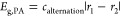
1where *c*_alternation_ is a constant, and the bond lengths for PA are defined in Figure 1b.

For π-conjugated polymers with more complex topologies than polyacetylene, such as those listed in Figure 1, the HOMO–LUMO level crossing was first described by Brédas^10,12^ and others^13,16^ demonstrating how the two frontier orbitals cross as a function of a BLA parameter defined as
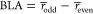
2where the bars specify the respective averages over the even and odd CC bond distances. Symmetries among bond distances may occur, and all bonds are included in the averaging. Following the numbering in Figure 1b, BLA > 0 describes an aromatic, and BLA < 0 a quinonoid structure.

This was a critical insight indicating the potential of polymers with quinonoid ground states as small bandgap polymers.^17−21^ This concept works well for many π-conjugated polymers, but not for all. For example, for the polymers in Figure 1a, such a BLA cannot be well-defined for the following two: PF, and BdiT. On the other hand, the nature of the topological phase, whether *Z*_2_ = 0 or 1, trivial or nontrivial, is uniquely definable for all π-conjugated polymers.^2,9,22^

It is essential to note that BLA is a computational parameter providing theoretical insights, but there is no external physical stimulus that can directly tune the BLA. In contrast, the presented level crossing as a function of external strain offers an experimentally well-defined transition between the nontrivial and trivial topological phases.

We undertook an extensive computational study subjecting the polymers listed in Figure 1 to positive (elongation, tensile) and negative (compression) uniaxial strain. The presentation of the resulting topological phase transition will be followed by an analysis of the transition with reasons for it to occur in this category of materials related to how the frontier orbitals react to the external stimulus of strain, which is defined as the change of the length of the repeat unit of the polymer (*l*) expressed in percent
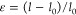
3where *l*_0_ is the equilibrium length of the unit cell measured parallel to the polymer chain direction.

The topological transition is confirmed by the presence of two phases, the nontrivial phase, or *Z*_2_ = 1 phase, and the trivial phase, or *Z*_2_ = 0 phase on either side of the transition. The nontrivial phase (*Z*_2_ = 1) displays two localized states at the two ends of the polymer whose corresponding energy levels are in the middle of the bandgap, while the trivial phase, (*Z*_2_ = 0) has no such states. There are other tests to confirm the value of the Zak-number,^9^*Z*_2_, for a material, but for the purposes of this work, as for previous work, this definition is sufficient and is workable.^1−3,22^Figures S1–S16 show the presence or absence of edge states at zero external strain.

A curious issue is, what determines whether the topological transition occurs upon compression or elongation. We will be able to tie this question to the zero-strain structure of the polymer, whether it is intrinsically aromatic or quinonoid. A particularly important question to address is how to obtain materials for which the transition occurs at smaller strain values. Since the transition occurs as a function ε at the strain value when the two frontier orbitals cross, their difference, the bandgap, *E*_g_(ε), plays a central role in the analysis and discussion.

## 2 Results and Discussion

**Figure 4:**
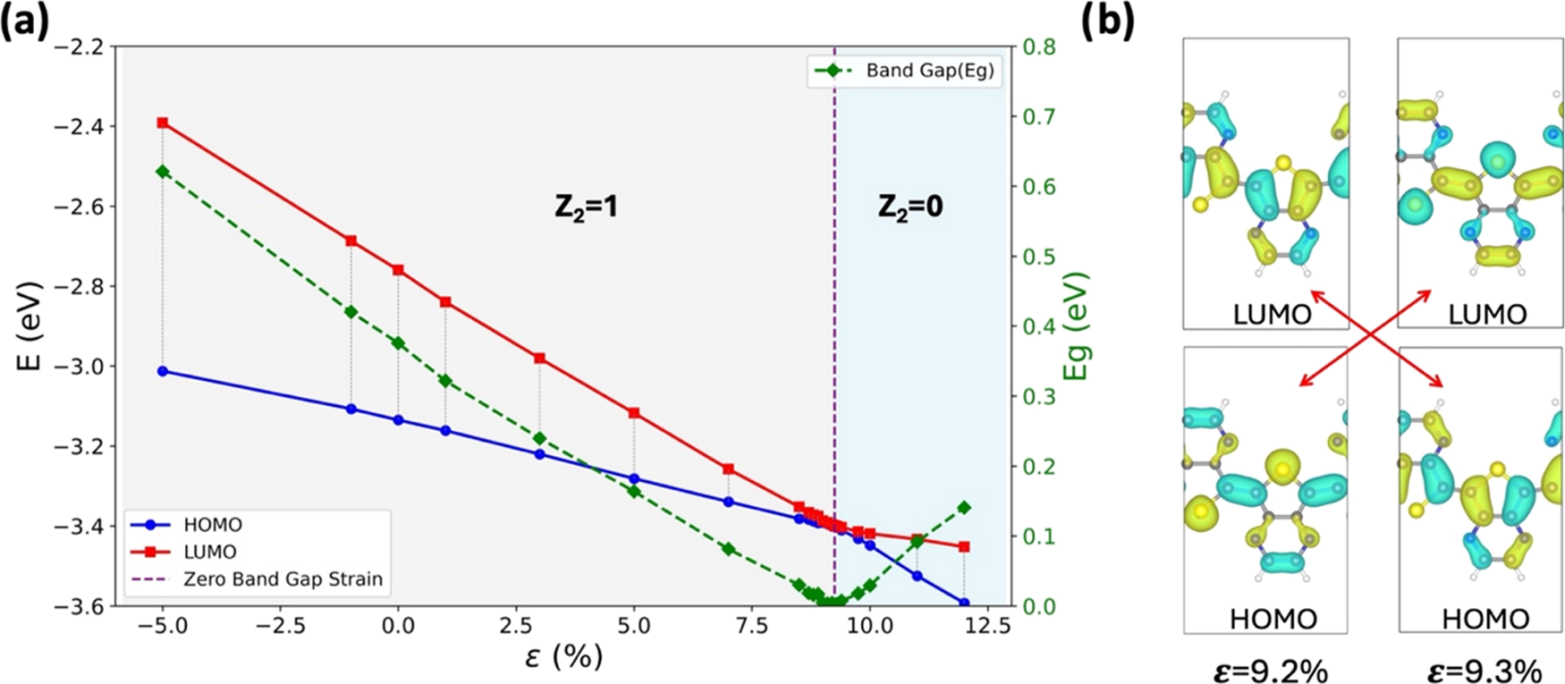
(a) Effect of uniaxial strain (ε) for the TPz π-conjugated polymer on the bandgap (in green), the highest occupied orbital (HOMO, in blue), and the lowest unoccupied orbital (LUMO, in red). The topological transition occurs near ε = 9.25% strain indicated by the vertical dashed line separating the *Z*_2_ = 1 region (nontrivial phase) from the *Z*_2_ = 0 region (trivial phase). (b) Level crossing at the topological transition for the TPz polymer between ε = +9.2% (nontrivial phase) and ε = +9.3% (trivial phase) tensile strain. At the transition the orbital phase changes at the CC bond connecting the unit cells (denoted as *r*_1_ in Figure 1b): the HOMO is in phase at *Z*_2_ = 1 and it is out of phase at *Z*_2_ = 0.

The strong coupling between uniaxial strain and the energy levels is the starting point for finding a topological transition for any given composition in this class of 1D polymers. This effect is somewhat counterintuitive, given that the repeat unit’s chemical composition is constant during the variation of strain and presumably all bonds along the polymer chain expand/shrink as the strain applied is tensile/compressive. Important to note that strain is an external parameter, and the strong dependency of orbital energy levels on strain is not guaranteed, as opposed to the nonphysical perturbation of bond length alternation, which in many cases directly leads to a nearly linear change in the bandgap as discussed above. Figure 4 illustrates the effect of this coupling on the bandgap and related orbital energy levels for the TPz polymer which represents an important example. In this case, at zero strain (ε = 0) the polymer is in a *Z*_2_ = 1 (nontrivial) phase that is identified as the quinonoid structure.

Focusing on the *r*_1_ bond in this case of a quinonoid system, the HOMO is bonding at zero strain, and upon the introduction of tensile strain, as this bond becomes longer, this level is expected to rise. On the other hand, the expectation would be that because the LUMO is antibonding (has an extra node at the *r*_1_ bond) it would be less destabilized upon the lengthening of this bond, and the LUMO level would decrease. Unfortunately, this simplified picture is not entirely correct, and in fact, both levels decrease, but the LUMO decreases more, as shown in Figure 4a. The complexity arises from the fact that all bonds adjust to the strain to some degree, and the level crossing is the result of these changes that do not cancel each other in general.

The central issue of this work is to determine the strength of this coupling for a good diversity of polymers represented in Figure 1a. The strength of this coupling in turn determines the strain value necessary to obtain a virtually zero bandgap at the topological transition point where the structure switches from the trivial to the nontrivial state, or vice versa.

We discuss the results as follows.

(a) Trivial vs nontrivial states in the TPz case.
(b) Identification of A vs Q ground states at ε = 0% strain for all polymers listed in Figure 1.
(c) Resulting coupling between strain and the bandgap for all polymers.
(d) This is followed by their analysis that is further aided by a newly introduced figure of merit targeting the efficiency at which strain can reduce the bandgap.
(e) Choice of a copolymer with a very small bandgap based on the insights learned.

**Figure 5:**
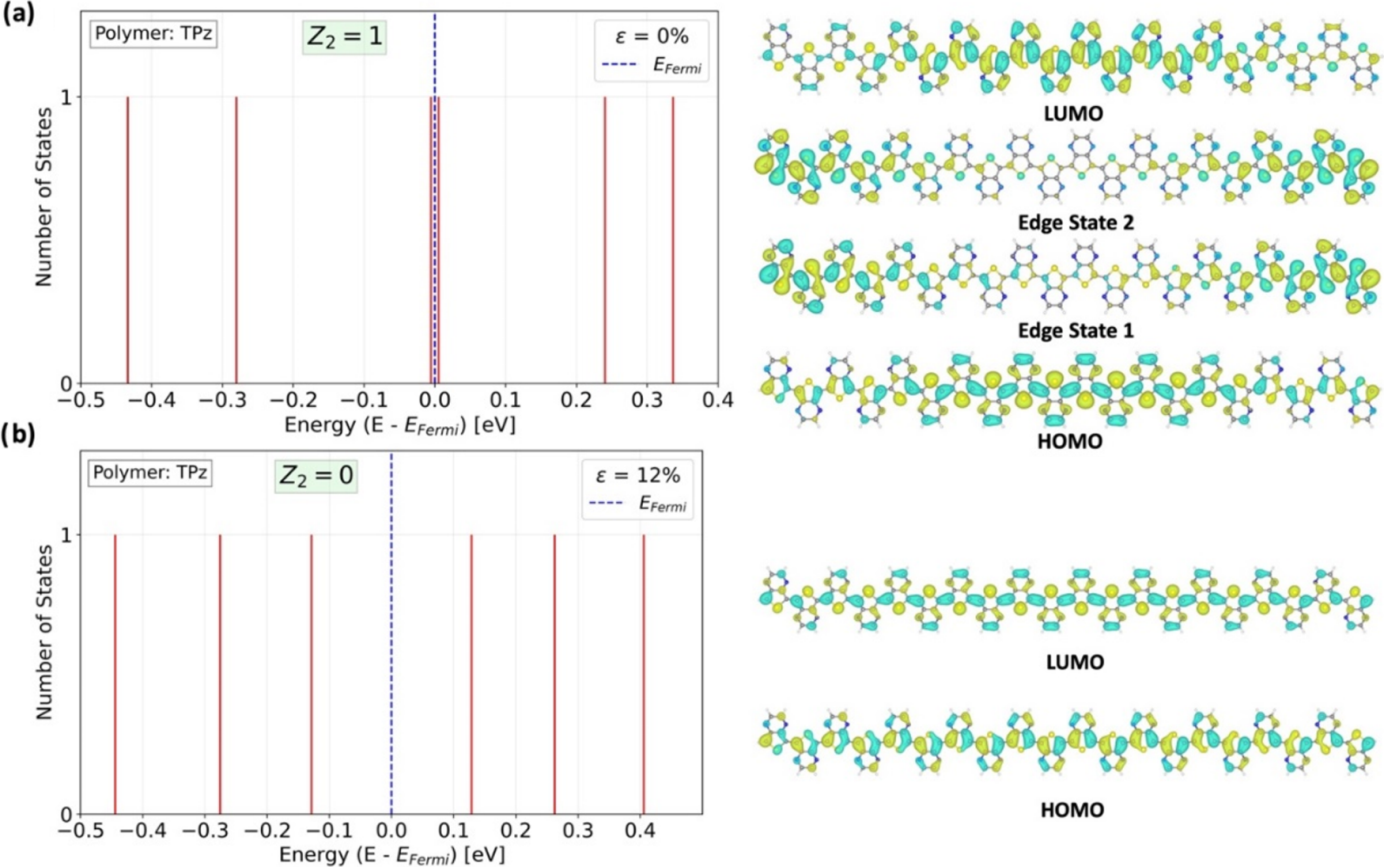
Nontrivial (a) and trivial (b) states of TPz at different strain values. The two degenerate edge states in (a) are illustrated as delocalized over both ends. The density of states is symbolic since there are continuous levels below the HOMO and above the LUMO, which are replaced by a finite *k*-point grid. The HOMO is bonding (in-phase) between the repeat units in the nontrivial case, while it is antibonding (out of phase) in the trivial case. The reverse situation is found for the LUMO wave functions.

The general analysis of the trivial vs nontrivial states of a conjugated polymer has been well established to show, that while in the trivial phase, all electronic states (wave functions) are delocalized, in the nontrivial state two localized spin states arise in the vicinity of the two termini.^1−3,9^ These localized end states are used in this work as the signature feature of the nontrivial (*Z*_2_ = 1) phase; their absence characterizes the trivial phase (*Z*_2_ = 0). The nontrivial phase is illustrated for the TPz polymer at ε = 0% in Figure 5a, while Figure 5b displays the lack of such terminal spin states at a high ε = 12% strain. The two edge states are degenerate and represent a single peak in the density of states in the middle of the bandgap at the Fermi level. (Due to numerical approximations, there is a very small splitting between these two states as depicted in Figure 5a, see comment in Section 4). The change of strain switched the phases of the orbitals at the *r*_1_ bond from bonding (no node) to antibonding (node) right at the transition between the nontrivial and trivial phases.

In this work, the identification of the trivial vs nontrivial topological phase in π-conjugated polymers relies on the presence or absence of these edge states shown in Figure 5 for TPz. In Figures S1–S16 we demonstrate that this is the case in general. In all cases we see a perfect correlation: all aromatic polymers belong to a *Z*_2_ = 0 (trivial) phase, and all quinonoid ones to a *Z*_2_ = 1 (nontrivial) phase based on the definition that relies on the bonding/antibonding phases of the HOMO wave function at the intercell carbon–carbon link as seen in Figure 3.

As we follow the transition continuously in Figure 4 the transition occurs at a specific strain value (9.25% for the polymer TPz). This effect results from the level crossing between the HOMO and LUMO at the topological transition point.^2,22^ A dramatic consequence is that at the transition point the computed bandgap becomes zero. A similar result was obtained for PITN albeit at a larger strain as illustrated in Figures S17–S19.

**Figure 6:**
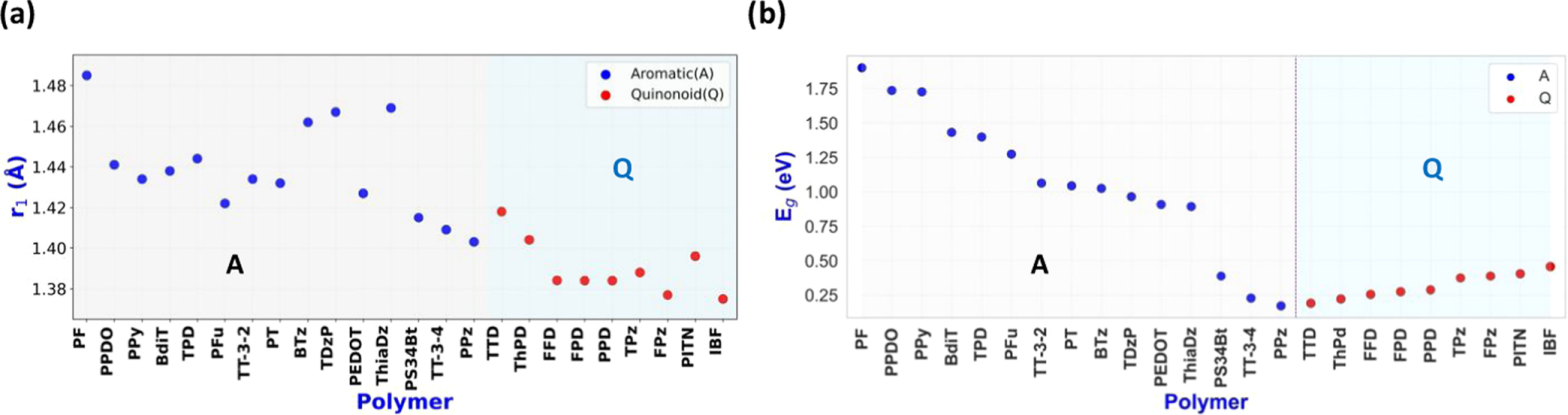
(a) Zero strain equilibrium linker CC bond lengths, left for the aromatic, and right for the quinonoid group of polymers. See Figure 1 for the identifiers of each polymer repeat unit. (b) Zero strain bandgap values for the presented polymers. The aromatic ones are shown by blue dots, the quinonoid ones by red dots. Note that some of the aromatic and all of the quinonoid ground state polymers show small bandgaps. The order of the polymers is the same in (a, b).

A critical issue, therefore, is the starting bandgap at ε = 0% strain, since the transition point between the trivial and nontrivial phases is where the two frontier levels cross. For this reason, a small starting bandgap appears to be one of the two factors to achieve the transition with relatively smaller strain values. Figure 6b displays these zero strain bandgap values. BLA parameters are listed in Figure S20 where all polymers except for PF and BdiT comply with the general trend that BLA > 0 describes an aromatic, and BLA < 0 a quinonoid structure. The exceptions are due to the lack of a well- defined single alternating chain of carbon atoms when rings are present in the sp^2^ network along the polymer chain.

Note that there is no simple back-of-the-envelope method by which these values can be qualitatively obtained although BLA and *E*_g_ correlate somewhat as shown for all investigated systems in Figure S21. As pointed out earlier,^13^ the bandgap is a result of the topology, the perturbing effects of the heteroatoms, and the geometry relaxation of the various bonds, eventually determining the A vs Q ground state structure. Since the *r*_1_ interunit carbon–carbon bond distances correlate with the aromatic vs quinonoid character of the ground state of the polymers shown in Figure 6a, there is a qualitative correlation between the overall order of bandgaps listed in Figure 6b and *r*_1_. Systems in the middle of the *r*_1_ value around 1.40–1.42 Å in the aromatic group, and all systems in the quinonoid group offer the smallest starting ε = 0% bandgaps. The shortest linker bonds correspond to Q ground state structures, while the longest ones to A structures. However, in the middle range around 1.40–1.42 Å, the *r*_1_ bond distance alone does not determine the nature of the ground state. In this range the topological definition based on the value of the Zak number (*Z*_2_) is determinative.

To answer the central question of this work, we introduce the strength of the coupling between the bandgap and strain, κ(ε), defined as
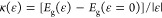
4where *E*_g_(ε) = *E*_LUMO_ – *E*_HOMO_ is the bandgap at strain ε (in %).

**Figure 7:**
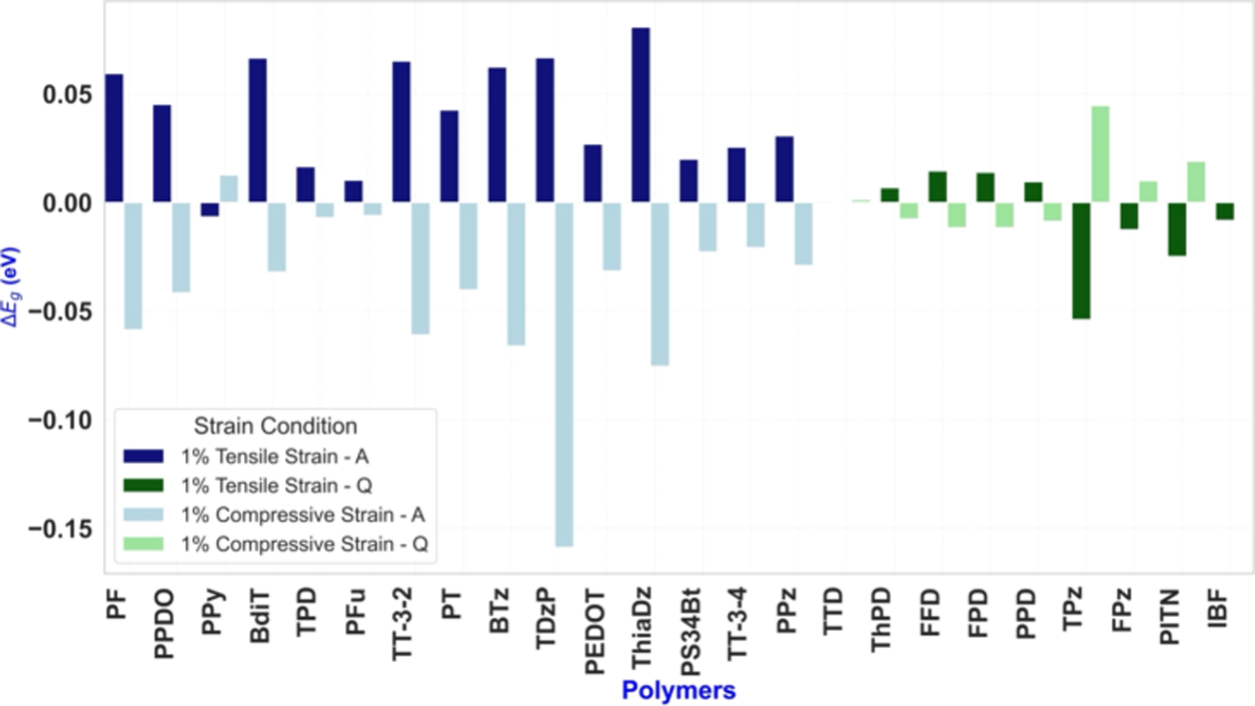
Change of bandgap, Δ*E*_g_ = *E*_g_(ε) – *E*_g_(ε = 0), corresponding to ε = ± 1% for all polymers discussed, in the order as listed in Figure 6.

κ(ε) values at 1 and 5% strain were computed for all polymers listed in Figure 1. The corresponding coupling values are given in Figure 7 for ε = ±1%. Figure S23 contains the respective values at ε = ±5% strain. The strength of the coupling varies widely. The trends at ε = ±5% strain are very similar to the trends at the smaller ε = ±1% strain values. Hence, we discuss only the couplings at ±1% strain.

Generally, the magnitude of the coupling is similar in absolute value but opposite in sign for expansion and compression. This is a consequence of the HOMO and LUMO levels depending approximately linearly upon strain. This is the case of TPz as seen in Figure 4, and for all other polymers discussed. Further examples are shown in Figure S25.

The trend for aromatic systems (PPz and others listed to the left of PPz in Figure 7) is that the bandgaps decrease upon compression and increase upon expansion; PPy is the only exception. For the quinonoid systems, we see one polymer with a very small coupling value (ThPD), and then four systems that behave like the aromatic systems. The rest of the quinonoid polymers display a trend opposite to that of the majority of the aromatic systems whereby their bandgaps increase upon compression and decrease upon expansion. Since the goal is to reduce the bandgap upon the external stimulus of tensile strain, these four are the most interesting and important ones. The sign of κ(ε = 1%) shows the direction in which the topological transition can be attained, and likewise, the sign of the applied strain for obtaining very small bandgaps.

We introduce a figure of merit, *m*(ε), that expresses the combination of the coupling, κ, with the starting bandgap, *E*_g_(ε = 0), in such a fashion that *m*(ε) increases when the coupling is strong and also increases when the starting bandgap is small. This figure of merit is large when a smaller strain value is expected to be necessary for achieving the topological phase transition.
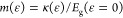
5

**Figure 8:**
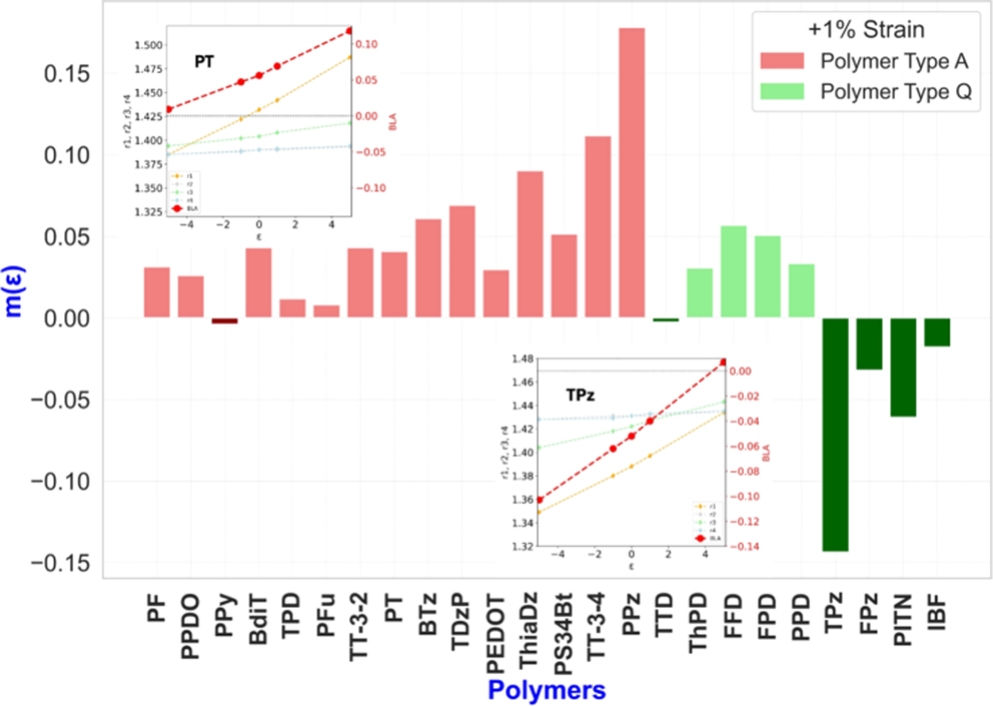
Figure of merit, *m*, as defined by eq 5 for all polymers in Figure 1 at the tensile strain ε = +1%. The polymers are listed from left to right in the same order as in Figure 6. The inserts show bond distances and the BLA as a function of strain for two typical cases, PT and TPz. Others are shown in Figure S22.

The respective *m*(ε) values are shown in Figure 8 for 1% tensile strain and Figure S24 contains the values corresponding to 5% strain. Since the 5% values are very similar, we discuss only the former. As a practical matter, there are three categories arising from the figure of merit. Small values refer to the relatively unresponsive polymers, whether *m*(ε) is positive or negative. Most interesting are polymers with the larger negative *m*(ε) values indicating their propensity to allow small or even very small bandgaps to be reached by tensile strain. Remarkably, all fall in the quinonoid category.

The largest positive value of the figure of merit belongs to PPz with the largest predicted relative optical shift (blue in this case) upon external strain of all the polymers investigated. TPz is second and both should be experimentally tested for the increase (PPz) or reduction (TPz) of the bandgap upon the application of tensile strain. It was unexpected that such a relatively minor difference in the polymer’s structure would lead to a major difference in their physical properties.

Upon further analysis, as discussed below, the effect can be rationalized. For all aromatic polymers, except for PPy, the bandgaps increase with tensile strain, which is a practically realizable external stimulus. This trend agrees with the one found before in the case of ethynylene bridged [n]acene polymers^22^ and other aromatic polymers^23^ and is related to the fact that the orbital phases of the HOMO is antibonding (out of phase) for the bond designated as *r*_1_ in Figure 1b. This leads to a lowering of the HOMO energy level while the opposite would be expected to occur for the LUMO level leading to a possible increase in the bandgap. The full analysis depends on all bonds in the structure, and therefore exceptions occur, but the general trend is an increase of the bandgap. Figure S25 displays the detailed origin of the behavior of the BLA as a function of strain. BLA changes significantly in most cases, some are illustrated in Figure S22 and the insets in Figure 8.

The typical aromatic BLA is shown for PT which is increasing with increasing tensile strain as one would find on intuitive grounds, based on the softer force constant of r_1_, the intercell carbon–carbon bond length. Starting with a positive BLA at ε = 0 leads to an increasing gap with increasing ε. For quinonoid polymers, the roles of the HOMO and LUMO are reversed as far as the phases at *r*_1_ are concerned leading to the expectation of a decrease of the bandgap upon tensile strain. The starting BLA is negative at ε = 0 and it increases with increasing ε, reducing the absolute value of the BLA, resulting in a smaller bandgap as seen for TPz. Here we see four exceptions to this trend^22^ listed between TTD and PPD in Figure 8. One of these, FPD, is listed in Figure 1b indicating a bond length alternation pattern. In general, while the BLA parameter is useful in sorting the behavior of the gap, these exceptions show that the coupling depends on the intricate details of topology and heteroatoms. This variation of responses offers the opportunity to find systems that potentially present even larger couplings than the ones analyzed here. The larger the coupling, the more potentially useful the system can be in applications where strains reduce the bandgap efficiently with reachable very small bandgaps upon stretching. The key point to find trends is whether the applied strain moves the electronic structure toward or away from the transition point. For some (PPy and especially TTD), the coupling is weak, and strain is not effective to move the electronic structure away or toward the topological transition. The change of BLA is relatively small, explaining the weak coupling in that case. For the typical aromatic polymer the reduction of the gap is achieved by compressive strain, and for TPz and a few other quinonoid π-conjugated polymers it is tensile strain.

**Figure 9:**
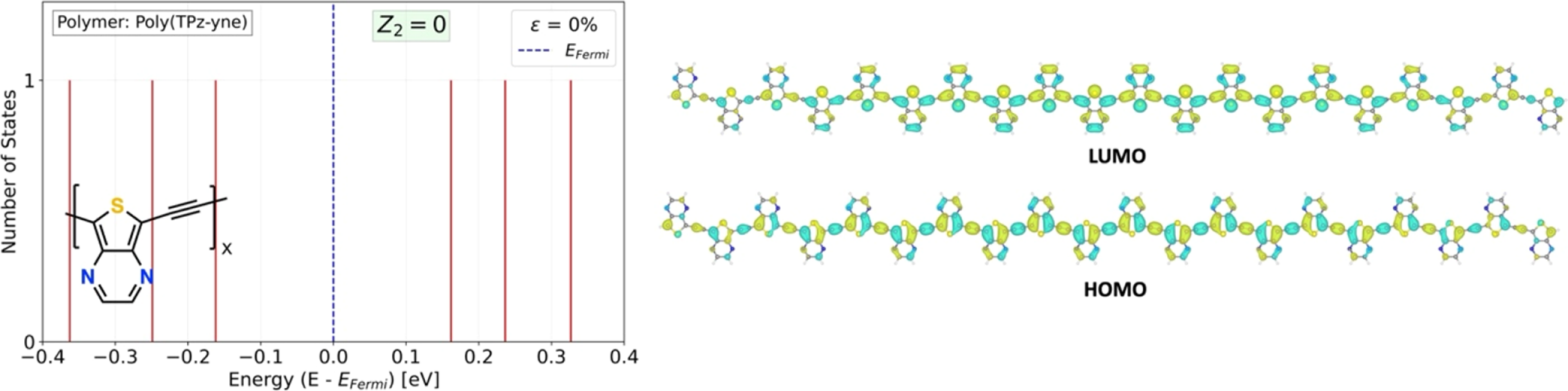
Trivial phase of a copolymer of TPz, poly(TPz-yne) at zero external strain. The resulting polymer is aromatic (trivial topological phase), but it appears near the topological phase transition point and therefore has a small bandgap. The chemical repeat unit is shown which is half of the full translational unit cell.

Further opportunity for designing small gap polymers is offered by the insights obtained by considering the topological phases of π-conjugated polymers. Starting with TPz at zero strain, which is one of the small bandgap *Z*_2_ = 1 polymers, a copolymer^8^ with alternating alkyne groups has the structural formula shown in Figure 9. The alkyne group is the most strongly alternating group, and one would anticipate that its insertion into the TPz polymer will push the structure in the *Z*_2_ = 0 aromatic direction. Indeed, as shown in Figure 9, the terminal states at the Fermi level have disappeared compared to those of TPz. However, the resulting bandgap of poly(TPz-yne) has the smallest value of all systems discussed here at 0.16 eV, which is ∼0.01 eV lower than the next lowest bandgap system, PPz. This type of construction of copolymers can lead the way toward polymers near the topological transition point with very small bandgaps even without external strain.

## 3 Conclusions

The presented computational results show that with proper application of uniaxial strain, quasimetallic states of π-conjugated polymers might be achieved near the topological transition point between their trivial and nontrivial phases. This fundamental insight can lead to potentially useful applications in some areas of molecular electronics, optoelectronics, organic solar cells, and quantum information technology.^2^

A broad range of π-conjugated polymers were considered and they represent a wide variety of responses to external strain as far as their electronic structures and bandgaps are concerned. The energy levels depend nearly linearly on the external strain, and therefore the response is for most cases very close in absolute value and opposite in sign for tensile and compressive strain. Some polymers display a strong dependency on the HOMO and LUMO energy levels, and also their difference changes significantly upon strain; these are the most interesting and promising for applications. Generally aromatic polymers increase their bandgaps with tensile strain, and only quinonoid polymers decrease it. While theoretically of interest to consider compressive strain to show a rather symmetrical behavior of this response with respect to the sign of strain, we find a group of quinonoid polymers that respond to the tensile strain with a significant reduction of the bandgap. This response has been characterized by a figure of merit introduced to describe quantitatively the propensity of a π-conjugated polymer to make it possible to achieve a very small bandgap upon the application of external strain to the polymer.

The analysis also provided a tool to exactly categorize a π-conjugated polymer as aromatic or quinonoid based on their Zak invariant: *Z*_2_ = 0 for aromatic and *Z*_2_ = 1 for quinonoid, the latter corresponding to the nontrivial topological phase with the presence of localized end states for the latter, and their absence for the former.

## 4 Methods

Computations have been performed at the PBE^24^ level of density functional theory (DFT) in the Quantum Espresso PWSCF v.7.1 program employing periodic boundary conditions (PBCs).^25^ PBE0^24,26^ computations were also performed for selected systems for validation purposes; for details see the SI section in Figure S32.

In the PBC calculations for polymers, the unit cells were aligned along the *x*-axis. In the other two directions, the unit cells were chosen to be large enough to avoid any significant interaction between periodic images. Similarly, in the isolated oligomer computations, the unit cell was chosen sufficiently large in all three directions to avoid interimage interactions. For quinonoid polymers, a very small splitting of edge states is observed, typically ranging from 10 to 30 meV. This splitting is due to the small interaction between the replicas in the periodic computation. The minimum distance between two hydrogens at 5.5 Å between the unit cells is far greater than the sum of the van der Waals radii. For the polymer computations, the shortest distance to the next atom in replica cells was even larger at ∼9 Å.

Two types of computations were performed:

1. Polymer computations generated optimized geometries, bandgaps, band structures, and orbital diagrams. For these, the unit cell along the polymer axis consisted of one chemical unit or, as appropriate, two chemical units which were placed in an anti configuration. These choices are listed in Table S1. Figure 1 displays the chemical repeat units. Then, these unit cells were subjected to uniaxial strain (ε). The geometry was optimized for each strain value.
2. For oligomer computations, the relaxed structures of the unit cells at the given ε strain were replicated along the polymer chain axis using the optimized lattice vector to build finite oligomers, with the ends terminated by hydrogens. Each finite chain was composed of an oligomer of 20 chemical repeat units to ensure that it was sufficiently long.

An ultrasoft pseudopotential^27^ was employed, with a kinetic energy cutoff of 140 Ry for all polymer computations whereas a smaller energy cutoff of 30 Ry was used for the finite-chain oligomers to make such computations tractable. Geometry relaxation was performed with an SCF convergence threshold of 1.0 × 10^–8^ au and a force convergence threshold of 1.0 × 10^–3^ au. Note that structure of PF polymer with 5% compressive strain becomes nonplanar. Marzari–Vanderbilt smearing of 0.02 Ry were applied for smooth SCF convergence.^28^ For all calculation dispersion correction were included through Grimme’s DFT-D2 empirical formalism.^29^ Unless specified otherwise, graphical representations of orbitals are given at the isovalues of 0.00005 au for the large isolated finite-chain and at 0.001 au for the unit cells. The 12 × 1 × 1 *k*-mesh has been used for the unit cell and single Γ-point computations were employed for the finite-chain oligomers.^30^ The overall reasonableness of the change of the total energy as a function of strain is reflected in the computed Young’s modulus values that are given in Figures S26–S31.

The terms HOMO and LUMO are used, although the computations refer to crystal orbitals based on periodic boundary conditions, referring to the terminology more familiar to the chemistry community. In the diagrams showing the number of states for different polymers, the Fermi energy is defined as
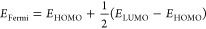
6

The figure of merit, *m*(ε), introduced in eq 5, can be used in a linear extrapolation to roughly estimate at which strain the transition would likely occur, see eq (S5) the SI section.

## Supporting Information Available

Lattice vectors; full names of the polymers; topological phases of all polymers at the equilibrium; strain-induced topological transition of PITN; bond length alternation (BLA) and bandgap (*E*_g_) correlation; change in bandgap and *m* values at ε = ± 5%; Young’s moduli; correlation between PBE and PBE0; torsional angle and topological phases (PDF)
Optimized coordinates (XYZ)

## Supplementary Material

ja4c10064_si_001.pdf

ja4c10064_si_002.xyz

## References

[ref1] Pendás A. M.; Contreras-García J.; Pinilla F.; Mella J. D.; Cardenas C.; Muñoz F. A chemical theory of topological insulators. Chem. Commun. 2019, 55 (82), 12281–12287. 10.1039/C9CC04054D. 31555782

[ref2] Cirera B.; Sánchez-Grande A.; de la Torre B.; Santos J.; Edalatmanesh S.; Rodríguez-Sánchez E.; Lauwaet K.; Mallada B.; Zbořil R.; Miranda R.; et al. Tailoring topological order and π-conjugation to engineer quasi-metallic polymers. Nat. Nanotechnol. 2020, 15 (6), 437–443. 10.1038/s41565-020-0668-7. 32313219

[ref3] González-Herrero H.; Mendieta-Moreno J. I.; Edalatmanesh S.; Santos J.; Martín N.; Écija D.; de la Torre B.; Jelinek P. Atomic Scale Control and Visualization of Topological Quantum Phase Transition in π-Conjugated Polymers Driven by Their Length. Adv. Mater. 2021, 33 (44), 2104495 10.1002/adma.202104495. 34536048

[ref4] Takeda Y.; Andrew T. L.; Lobez J. M.; Mork A. J.; Swager T. M. An Air-Stable Low-Bandgap n-Type Organic Polymer Semiconductor Exhibiting Selective Solubility in Perfluorinated Solvents. Angew. Chem., Int. Ed. 2012, 51 (36), 9042–9046. 10.1002/anie.201204066. 22887312

[ref5] Schmatz B.; Ponder J. F. Jr; Reynolds J. R. Multifunctional triphenylamine polymers synthesized via direct (hetero) arylation polymerization. J. Polym. Sci., Part A: Polym. Chem. 2018, 56 (1), 147–153. 10.1002/pola.28896.

[ref6] Kawabata K.; Saito M.; Osaka I.; Takimiya K. Very small bandgap π-conjugated polymers with extended thienoquinoids. J. Am. Chem. Soc. 2016, 138 (24), 7725–7732. 10.1021/jacs.6b03688. 27224874

[ref7] Leventis A.; Royakkers J.; Rapidis A. G.; Goodeal N.; Corpinot M. K.; Frost J. M.; Bučar D.-K. i.; Blunt M. O.; Cacialli F.; Bronstein H. Highly luminescent encapsulated narrow bandgap polymers based on diketopyrrolopyrrole. J. Am. Chem. Soc. 2018, 140 (5), 1622–1626. 10.1021/jacs.7b13447. 29337534

[ref8] Mikie T.; Osaka I. Small-bandgap quinoid-based π-conjugated polymers. J. Mater. Chem. C 2020, 8 (41), 14262–14288. 10.1039/D0TC01041C.

[ref9] Cao T.; Zhao F.; Louie S. G. Topological phases in graphene nanoribbons: junction states, spin centers, and quantum spin chains. Phys. Rev. Lett. 2017, 119 (7), 076401 10.1103/PhysRevLett.119.076401. 28949674

[ref10] Brédas J. L.; Heeger A.; Wudl F. Towards organic polymers with very small intrinsic band gaps. I. Electronic structure of polyisothianaphthene and derivatives. J. Chem. Phys. 1986, 85 (8), 4673–4678. 10.1063/1.451741.

[ref11] Kürti J.; Surján P. Quinoid vs aromatic structure of polyisothianaphthene. J. Chem. Phys. 1990, 92 (5), 3247–3248. 10.1063/1.457883.

[ref12] Brédas J. L. Relationship between band gap and bond length alternation in organic conjugated polymers. J. Chem. Phys. 1985, 82 (8), 3808–3811. 10.1063/1.448868.

[ref13] Kertesz M.; Choi C. H.; Yang S. Conjugated polymers and aromaticity. Chem. Rev. 2005, 105 (10), 3448–3481. 10.1021/cr990357p. 16218558

[ref14] Grover G.; Peters G. M.; Tovar J. D.; Kertesz M. Quinonoid vs. aromatic structures of heteroconjugated polymers from oligomer calculations. Phys. Chem. Chem. Phys. 2020, 22 (20), 11431–11439. 10.1039/D0CP00606H. 32386288

[ref15] Peierls R. E. Quantum Theory of Solids; Oxford University Press, 1955.

[ref16] Mintmire J.; White C.; Elert M. Heteroatom effects in heterocyclic ring chain polymers. Synth. Met. 1986, 16 (2), 235–243. 10.1016/0379-6779(86)90116-5.

[ref17] Wudl F.; Kobayashi M.; Heeger A. Poly (isothianaphthene). J. Org. Chem. 1984, 49 (18), 3382–3384. 10.1021/jo00192a027.

[ref18] Bérubé N.; Gaudreau J.; Côté M. Low band gap polymers design approach based on a mix of aromatic and quinoid structures. Macromolecules 2013, 46 (17), 6873–6880. 10.1021/ma401358r.

[ref19] Hayashi Y.; Kawauchi S. Development of a quantum chemical descriptor expressing aromatic/quinoidal character for designing narrow-bandgap π-conjugated polymers. Polym. Chem. 2019, 10 (41), 5584–5593. 10.1039/C9PY00987F.

[ref20] Kleinhenz N.; Yang L.; Zhou H.; Price S. C.; You W. Low-band-gap polymers that utilize quinoid resonance structure stabilization by thienothiophene: Fine-tuning of HOMO level. Macromolecules 2011, 44 (4), 872–877. 10.1021/ma1024126.

[ref21] Lee Y. S.; Kertesz M. The effect of additional fused rings on the stabilities and the band gaps of heteroconjugated polymers. Int. J. Quantum Chem. 1987, 32 (S21), 163–170. 10.1002/qua.560320719.

[ref22] Bhattacharjee R.; Kertesz M. Continuous Topological Transition and Bandgap Tuning in Ethynylene-Linked Acene π-Conjugated Polymers through Mechanical Strain. Chem. Mater. 2024, 36 (3), 1395–1404. 10.1021/acs.chemmater.3c02547. 38375000 PMC10876101

[ref23] Cachaneski-Lopes J. P.; Batagin-Neto A. Effects of mechanical deformation on the opto-electronic responses, reactivity, and performance of conjugated polymers: a DFT study. Polymers 2022, 14 (7), 1354 10.3390/polym14071354. 35406228 PMC9002523

[ref24] Perdew J. P.; Burke K.; Ernzerhof M. Generalized gradient approximation made simple. Phys. Rev. Lett. 1996, 77 (18), 3865 10.1103/PhysRevLett.77.3865. 10062328

[ref25] Giannozzi P.; Baroni S.; Bonini N.; Calandra M.; Car R.; Cavazzoni C.; Ceresoli D.; Chiarotti G. L.; Cococcioni M.; Dabo I.; et al. QUANTUM ESPRESSO: a modular and open-source software project for quantum simulations of materials. J. Phys.: Condens. Matter 2009, 21 (39), 395502 10.1088/0953-8984/21/39/395502. 21832390

[ref26] Barnes T. A.; Kurth T.; Carrier P.; Wichmann N.; Prendergast D.; Kent P. R.; Deslippe J. Improved treatment of exact exchange in Quantum ESPRESSO. Comput. Phys. Commun. 2017, 214, 52–58. 10.1016/j.cpc.2017.01.008.

[ref27] Vanderbilt D. Soft self-consistent pseudopotentials in a generalized eigenvalue formalism. Phys. Rev. B 1990, 41 (11), 7892 10.1103/PhysRevB.41.7892. 9993096

[ref28] Marzari N.; Vanderbilt D.; De Vita A.; Payne M. Thermal contraction and disordering of the Al (110) surface. Phys. Rev. Lett. 1999, 82 (16), 3296 10.1103/PhysRevLett.82.3296.

[ref29] Grimme S. Semiempirical GGA-type density functional constructed with a long-range dispersion correction. J. Comput. Chem. 2006, 27 (15), 1787–1799. 10.1002/jcc.20495. 16955487

[ref30] Monkhorst H. J.; Pack J. D. Special points for Brillouin-zone integrations. Phys. Rev. B 1976, 13 (12), 5188 10.1103/PhysRevB.13.5188.

