# Supplementary material for: Topological Transition in Aromatic and Quinonoid π-Conjugated Polymers Induced by Static Strain
Source: J Am Chem Soc. 2024 Sep 13;146(38):26497–504. doi: 10.1021/jacs.4c10064 (PMC11440520; doi:10.1021/jacs.4c10064)
Supplement: Supplementary file 1 — ja4c10064_si_001.pdf [file ja4c10064_si_001.pdf]

Supporting information section for

Topological transition in aromatic and quinonoid of π-conjugated polymers induced by static strain

Rameswar Bhattacharjee*, Miklos Kertesz*

Department of Chemistry, Georgetown University, Washington, D.C. 20057, United States.

Table of contents

1. Lattice vectors, Table S1.
2. Topological phases and edge states, Figures S1-S16.
3. Topological transition for PITN upon tensile strain,), Figures S17-S19.
4. Bond length alternation (BLA) and bandgap (E_g), Figures S20-S22.
5. Bandgap changes and figure of merit (m) at strain $\varepsilon = \pm$ 5 %, Figures S23-S24.
6. The nature of level crossing upon strain, Figure S25.
7. Young's modulus, Figures S26-S31.
8. Correlation between PBE and PBE0, Figure S32.
9. Introduction of torsion along the polymer chain, figures S33-35.
10. Using the figure of merit, m, to estimate the critical strain value.
11. References.

Emails:

Rameswar Bhattacharjee rb1820@georgetown.edu

Miklos Kertesz kertesz@georgetown.edu

1. Lattice vectors and name of polymers

Table S1: Optimized lattice vectors along the chain axis for all 24 polymers considered in this study at zero external strain

Polymer	Names of polymers	Number of chemical repeat units per unit cell	Length of the optimized lattice vector ($\lvert\vec{l}\rvert$) in Å
PF	poly(9H-fluorene)	2	16.908
PPDO	poly(pyrrolo[3,4-*c*]pyrrole-1,3(2*H*,5*H*)-dione)	2	7.296
PPy	poly(pyrrole)	2	7.175
BdiT	poly(benzo-1,2-b:4,5-b'-dithiophene)	1	8.476
TPD	poly(thieno[3,4-*c*]pyrrole-4,6(5*H*)-dione)	2	7.919
PFu	poly(furan)	2	6.967
TT-3-2	poly(thieno-3,2-[b]thiophene)	1	6.053
PT	poly(thiophene)	2	7.805
BTz	poly(2H-benzo[d][1,2,3]triazole)	2	8.895
TDzP	poly(1,2,5-thiadiazolo-3,4-[c]pyridine)	2	8.709
PEDOT	poly(3,4-ethylenedioxythiophene)	2	7.877
ThiaDz	poly(benzo-1,2,5-thiadiazole)	2	8.931
PS34Bt	poly(selenopheno[2,3-c]thiophene)	2	7.856
TT-3-4	poly(thieno-3,4-[b]thiophene)	2	7.865
PPz	poly(6H-pyrrolo[3,4-b]pyrazine)	2	7.188
TTD	Poly(thieno[3,4-c]thiophene-1,4-dione)	1	4.999

ThPD	Poly(thieno[3,4-c]pyrrole-1,4-dione)	1	4.896
FFD	Poly(furo[3,4-c]furan-1,4-dione)	1	4.784
FPD	Poly(furo[3,4-c]pyrrole-1,4-dione)	1	4.776
PPD	poly(2,5-dihydropyrrolo-3,4-[c]pyrrole-1,4-dione)	1	4.768
TPz	poly(thieno-3,4-[b]pyrazine)	2	7.831
FPz	poly(furo[3,4-b]pyrazine)	2	6.991
PITN	poly(benzo[c]thiophene)	2	7.875
IBF	poly(isobenzofuran)	2	6.993

2. The nature of the ε=0 phases: which polymer is in the Z_2=0 or Z_2=1 phase?

In the following figures we demonstrate the nature of the ε=0 strain-free phases by focusing on the frontier orbitals near the Fermi level, E_F. Presence of two levels near E_F indicate a Z_2=1 (non-trivial) phase; their absence shows this to be a Z_2=0 (trivial) phase. Four aromatic polymers (PF, PPDO, PPy, BdiT) and one quinonoid (TPz) are not listed. (The latter is discussed in the main text in detail.) In all cases we see a perfect correlation: all aromatic polymers belong to a Z_2=0 (trivial) phase, and all quinonoid ones to a Z_2=1 (non-trivial) phase based on the definition that relies on the bonding/antibonding phases of the HOMO wavefunction at the inter-cell carbon-carbon link as explained in the main text.

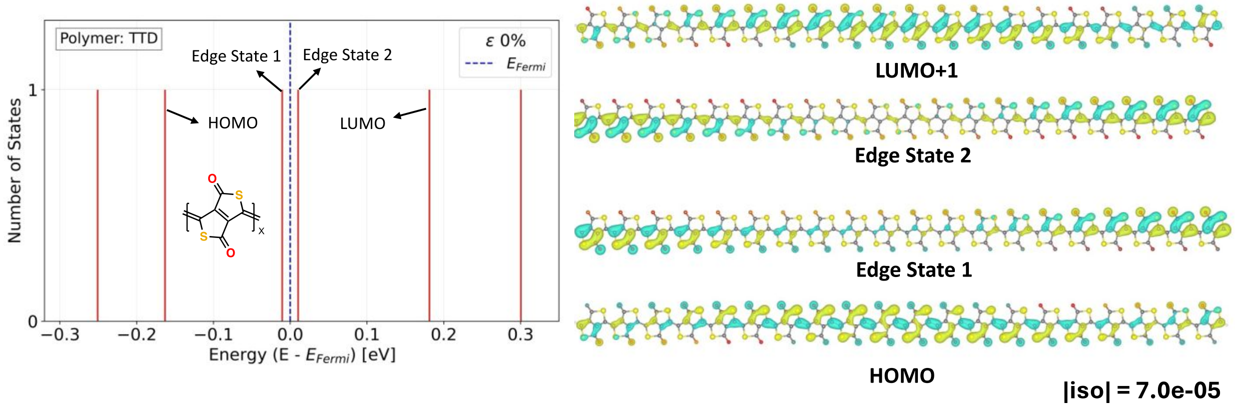

Figure S1: Non-trivial (Z_2=1) phase polymer TTD, which is quinonoid. The two degenerate edge states are shown as delocalized over both ends. The number of states is symbolic, with continuous levels below the HOMO and above the LUMO not being indicated here.

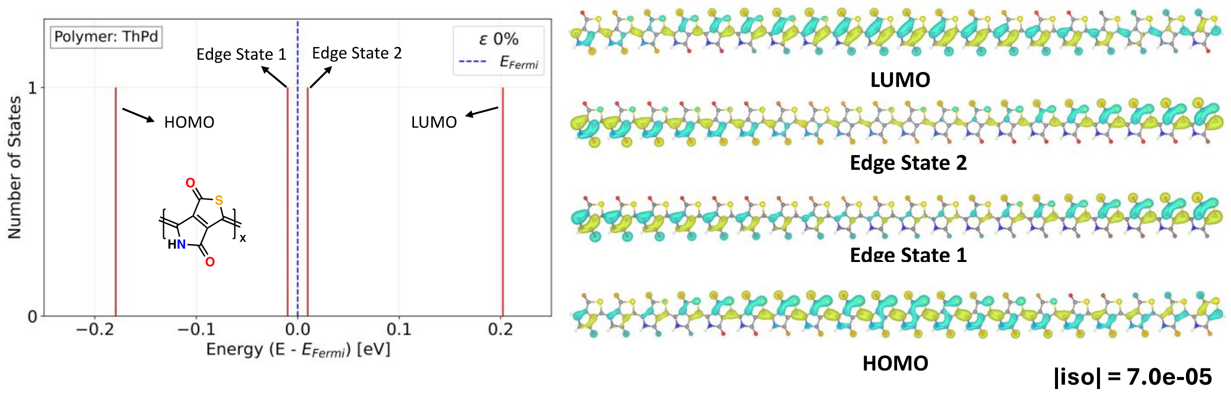

Figure S2: Non-trivial (Z_2=1) phase polymer ThPD, which is quinonoid. The two degenerate edge states are shown as delocalized over both ends. The number of states is symbolic, with continuous levels below the HOMO and above the LUMO not being indicated here.

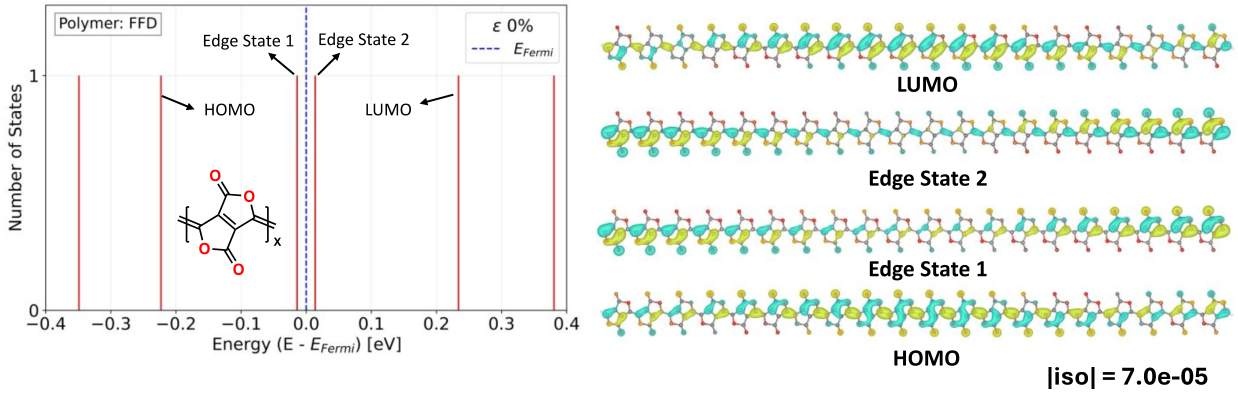

Figure S3: Non-trivial (Z_2=1) phase polymer FFD, which is quinonoid. The two degenerate edge states are shown as delocalized over both ends. The number of states is symbolic, with continuous levels below the HOMO and above the LUMO not being indicated here.

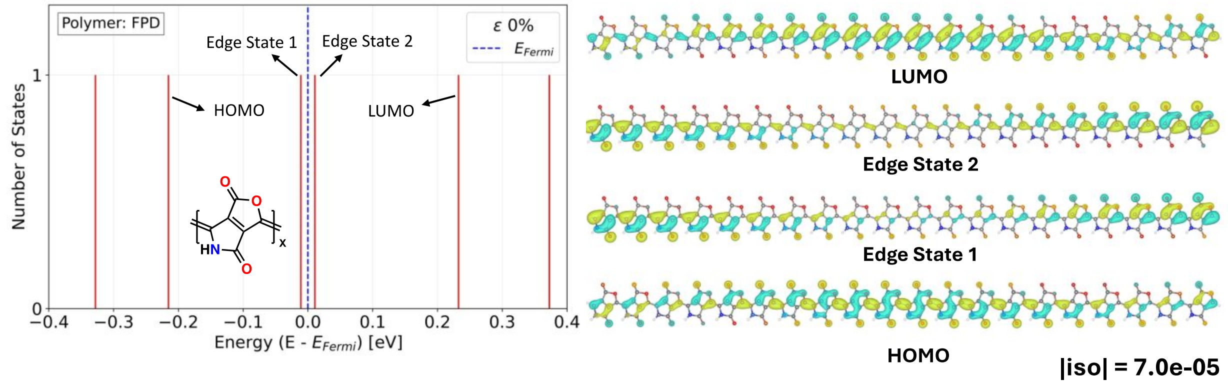

Figure S4: Non-trivial (Z_2=1) phase polymer FPD, which is quinonoid. The two degenerate edge states are shown as delocalized over both ends. The number of states is symbolic, with continuous levels below the HOMO and above the LUMO not being indicated here.

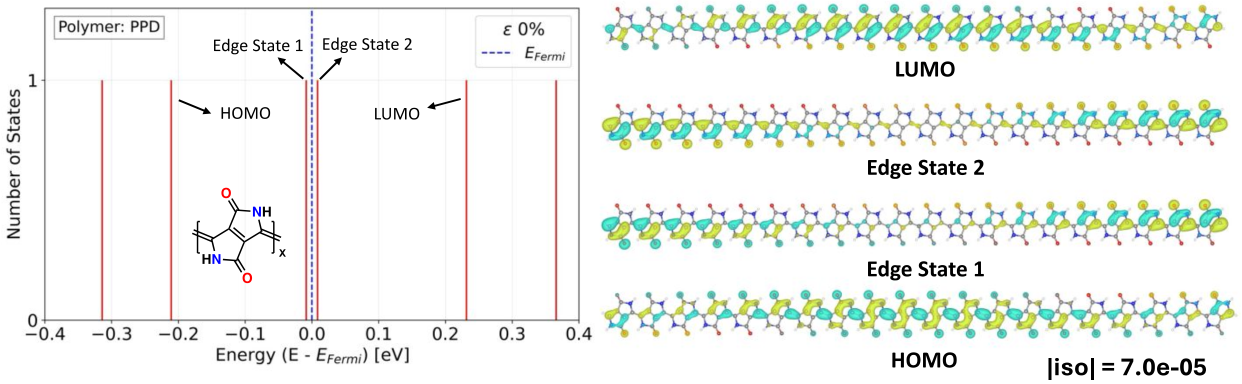

Figure S5: Non-trivial (Z_2=1) phase polymer PPD, which is quinonoid. The two degenerate edge states are shown as delocalized over both ends. The number of states is symbolic, with continuous levels below the HOMO and above the LUMO not being indicated here.

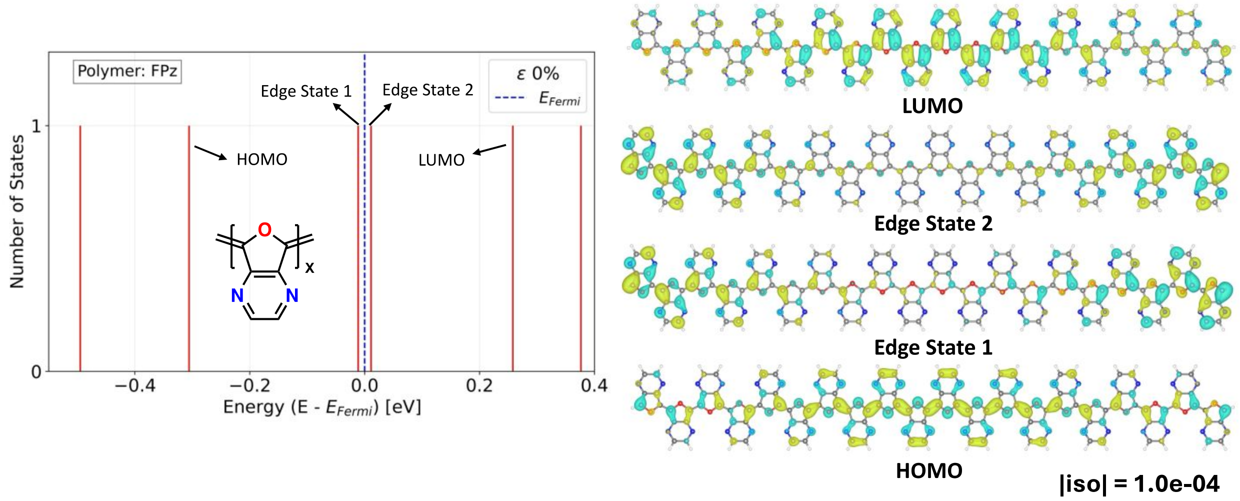

Figure S6: Non-trivial (Z_2=1) phase polymer FPz, which is quinonoid. The two degenerate edge states are shown as delocalized over both ends. The number of states is symbolic, with continuous levels below the HOMO and above the LUMO not being indicated here.

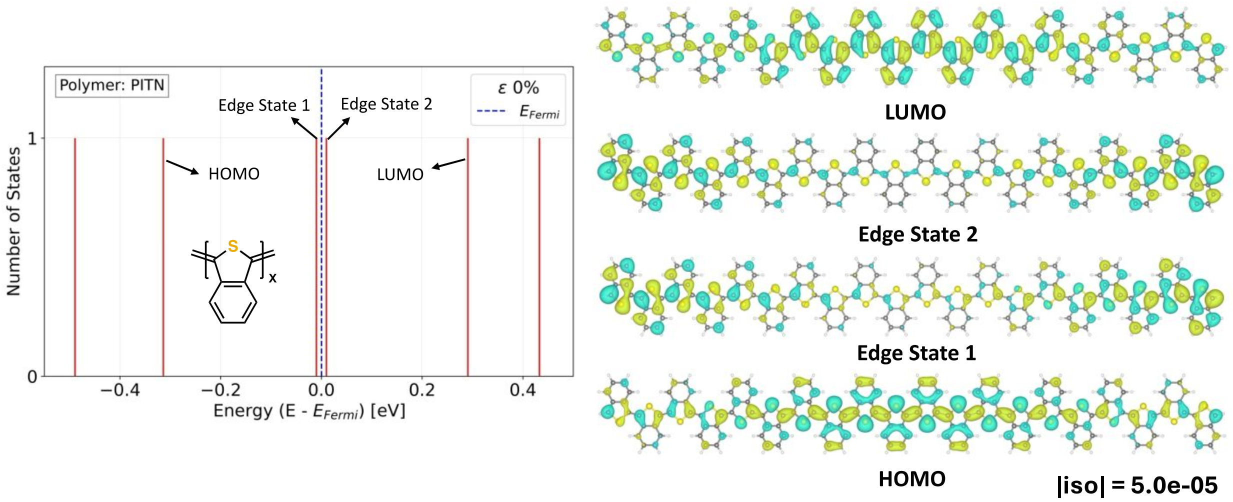

Figure S7: Non-trivial (Z_2=1) phase polymer PITN, which is quinonoid. The two degenerate edge states are shown as delocalized over both ends. The number of states is symbolic, with continuous levels below the HOMO and above the LUMO not being indicated here.

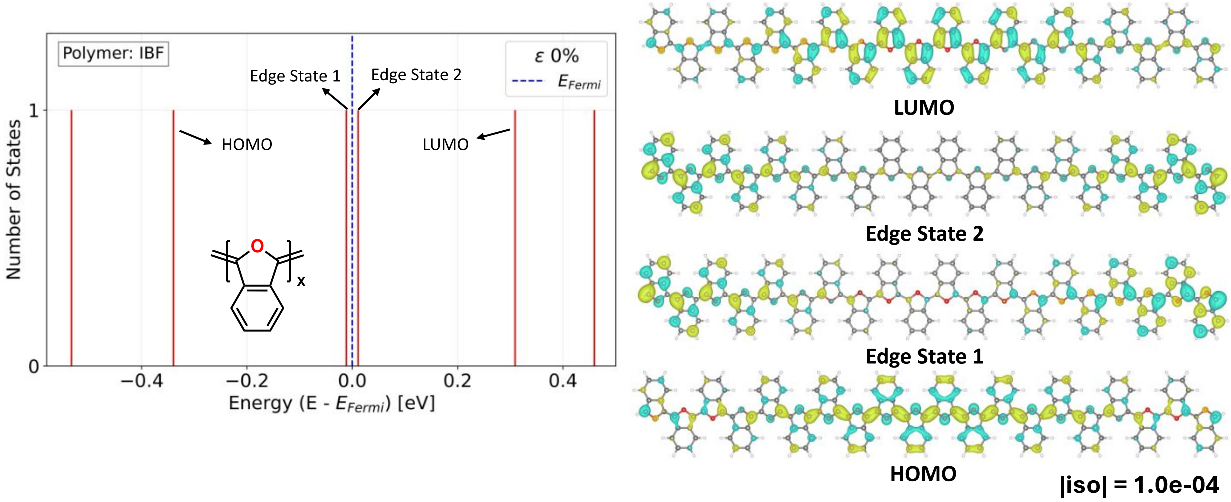

Figure S8: Non-trivial (Z_2=1) phase polymer IBF, which is quinonoid. The two degenerate edge states are shown as delocalized over both ends. The number of states is symbolic, with continuous levels below the HOMO and above the LUMO not being indicated here.

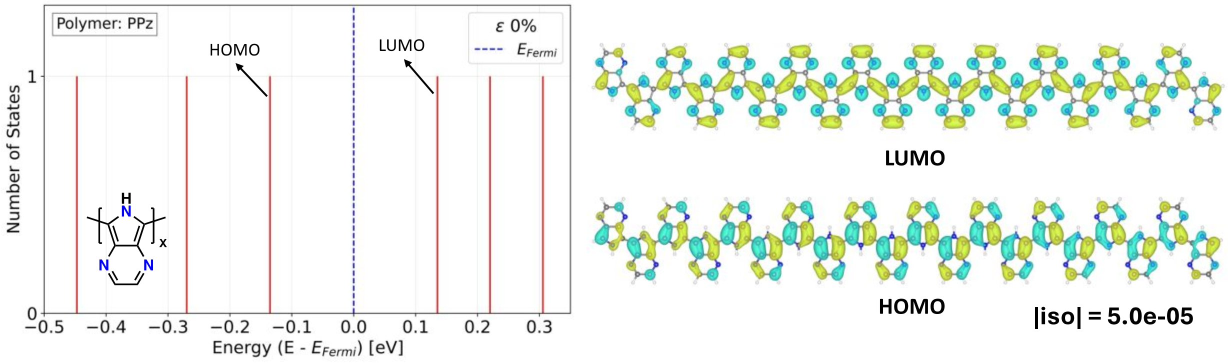

Figure S9: Trivial (Z_2=0) phase polymer PPz, which is aromatic. No edge state is observed for the polymer. The number of states is symbolic, with continuous levels below the HOMO and above the LUMO not being indicated here.

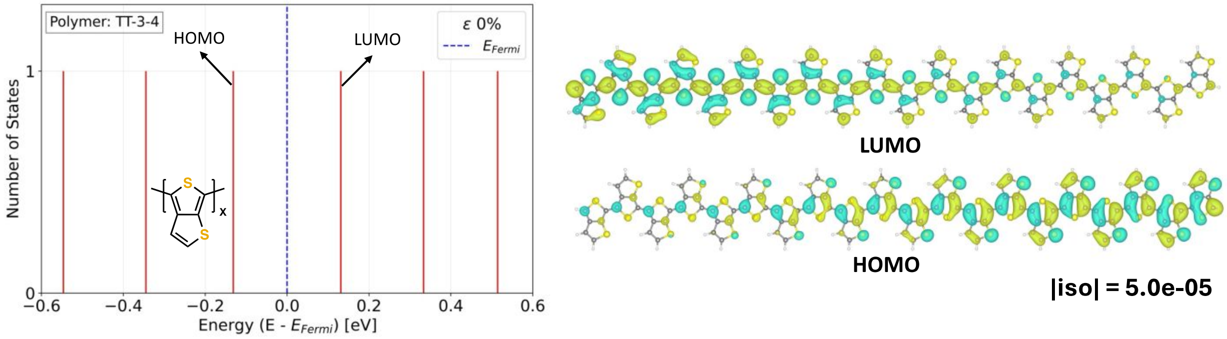

Figure S10: Trivial (Z_2=0) phase polymer TT-3-4, which is aromatic. No edge state is observed for the polymer. The number of states is symbolic, with continuous levels below the HOMO and above the LUMO not being indicated here.

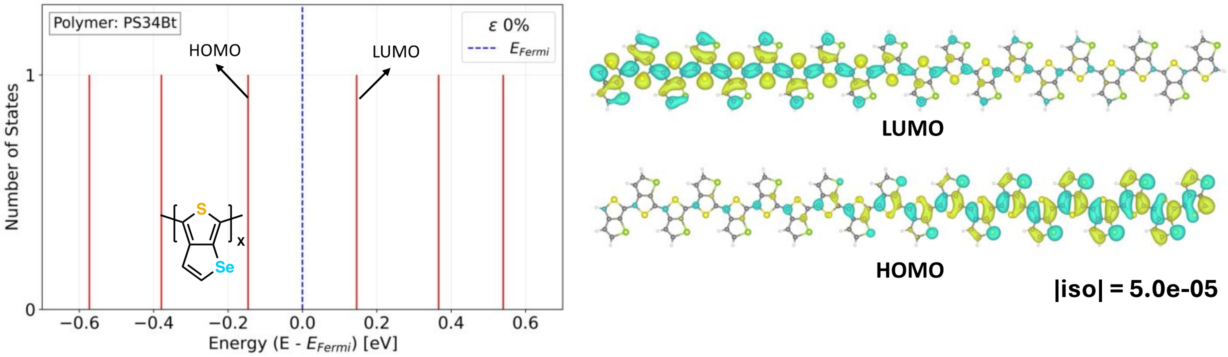

Figure S11: Trivial (Z_2=0) phase polymer PS34Bt, which is aromatic. No edge state is observed for the polymer. The number of states is symbolic, with continuous levels below the HOMO and above the LUMO not being indicated here.

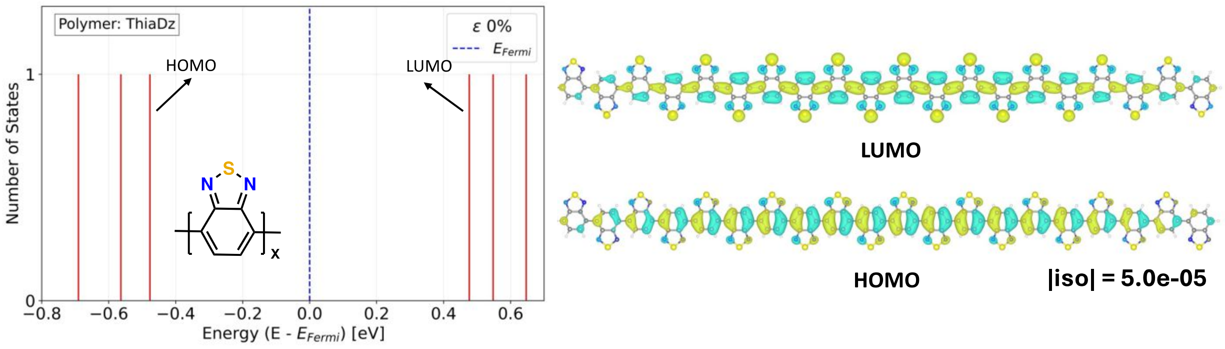

Figure S12: Trivial (Z_2=0) phase polymer ThiaDz, which is aromatic. No edge state is observed for the polymer. The number of states is symbolic, with continuous levels below the HOMO and above the LUMO not being indicated here.

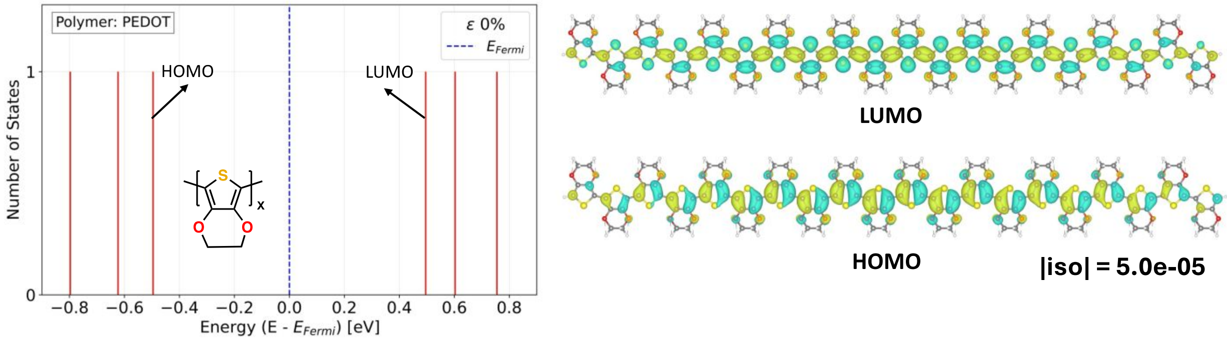

Figure S13: Trivial (Z_2=0) phase polymer PEDOT, which is aromatic. No edge state is observed for the polymer. The number of states is symbolic, with continuous levels below the HOMO and above the LUMO not being indicated here.

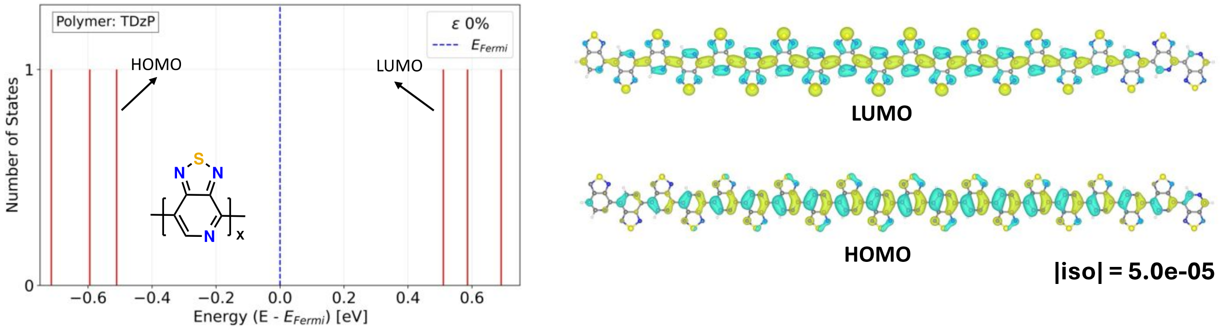

Figure S14: Trivial (Z_2=0) phase polymer TDzP, which is aromatic. No edge state is observed for the polymer. The number of states is symbolic, with continuous levels below the HOMO and above the LUMO not being indicated here.

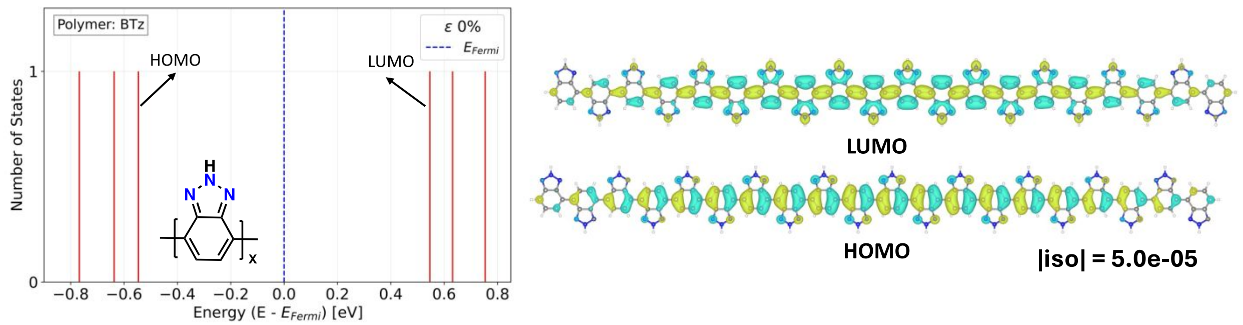

Figure S15: Trivial (Z_2=0) phase polymer BTz, which is aromatic. No edge state is observed for the polymer. The number of states is symbolic, with continuous levels below the HOMO and above the LUMO not being indicated here.

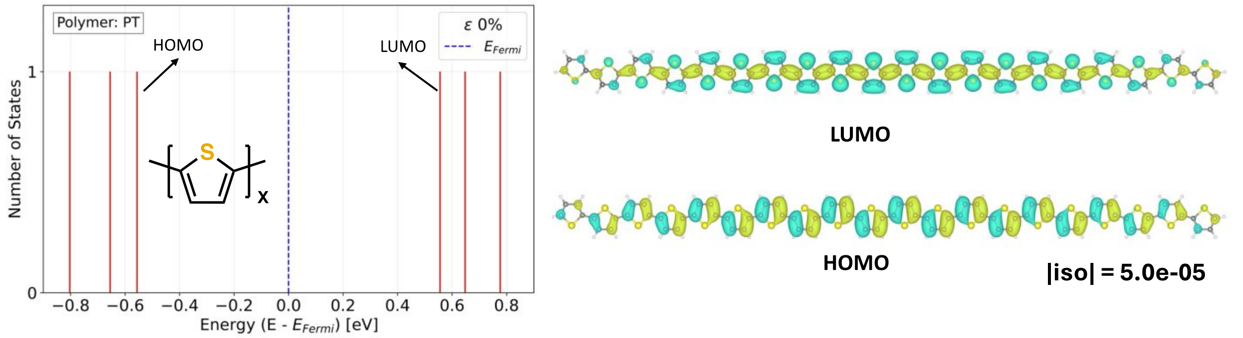

Figure S16: Trivial (Z_2=0) phase polymer PT, which is aromatic. No edge state is observed for the polymer. The number of states is symbolic, with continuous levels below the HOMO and above the LUMO not being indicated here.

3. Topological transition for PITN upon tensile strain

The HOMO, LUMO and the HOMO-LUMO gaps are shown in Figure S17a as a function of tensile strain. The level crossing occurs between 16% and 17% as illustrated in Figure S17b. The natures of the states (non-trivial below 16% and trivial above17%) are illustrated in Figures S18 and S19 with the former showing the presence of edge states and the latter the absence thereof, respectively.

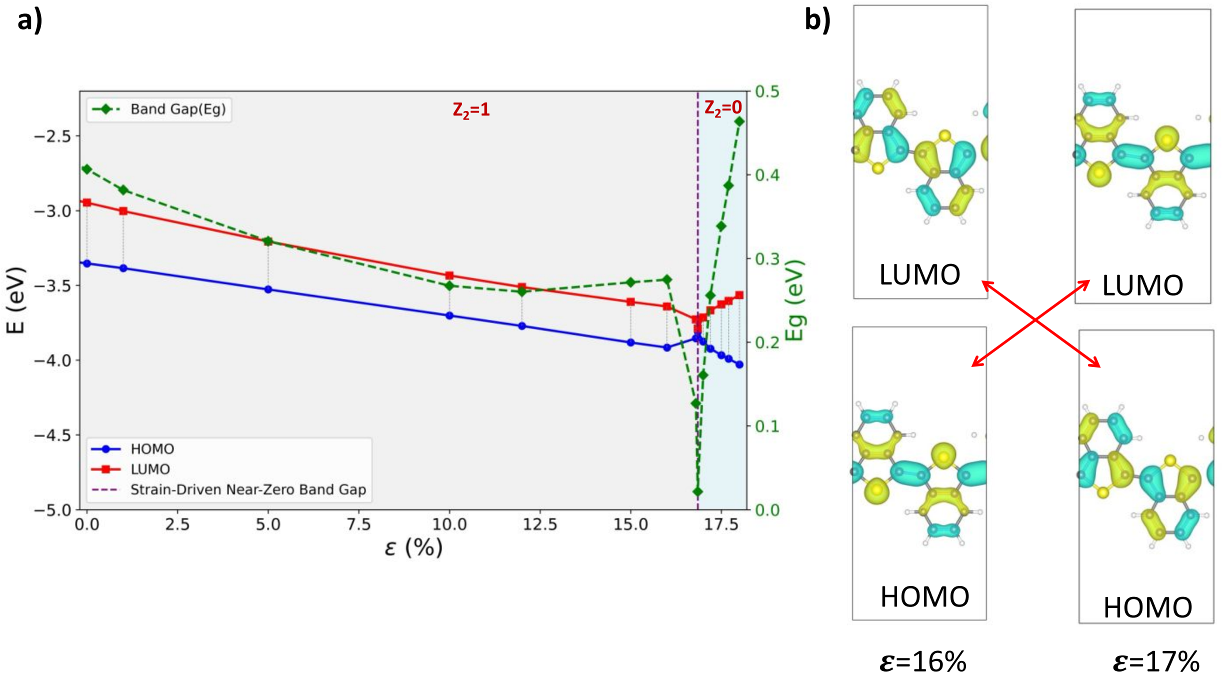

Figure S17: (a) The effect of uniaxial strain (ε) for the PITN π-conjugated polymer on the bandgap (in green), the highest occupied orbital (HOMO, in blue), and the lowest unoccupied orbital (LUMO, in red). The topological transition occurs near ε=16.85 % strain indicated by the vertical dashed line separating the Z_2=1 region (non-trivial phase) from the Z_2=0 region (trivial phase). (b) Level crossing at the topological boundary for the TPz polymer between ε=+16.0 % (non-trivial phase) and ε=+17.0 % (trivial phase) tensile strain. At the transition the orbital phase changes at the CC bond connecting the unit cells.

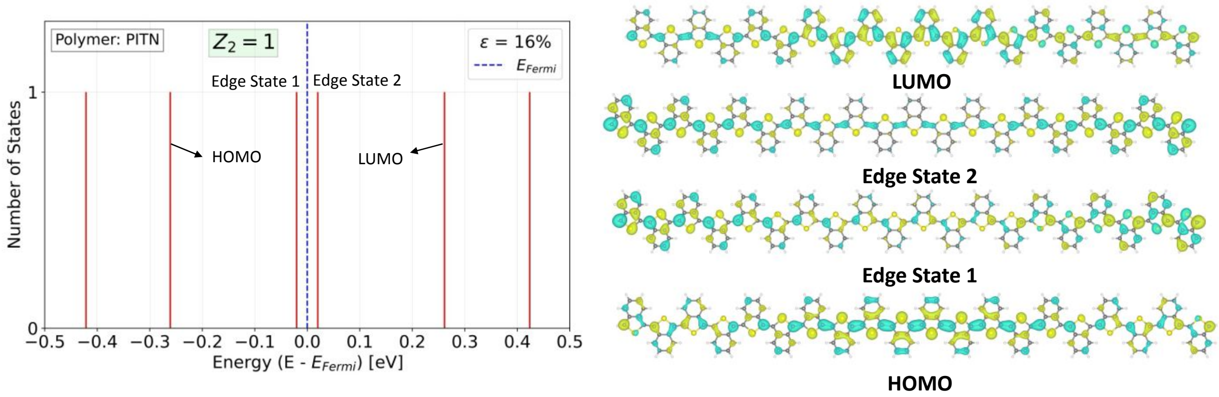

Figure S18: Non-trivial states of PITN at +16.0% strain values. The two degenerate edge states are illustrated as delocalized over both ends. The density of states is symbolic since there are continuous levels below the HOMO and above the LUMO, which are replaced by a finite k-point grid. The HOMO is bonding (in-phase) between the repeat units while LUMO is antibonding (out of phase). Isovalue = 0.00007 a.u.

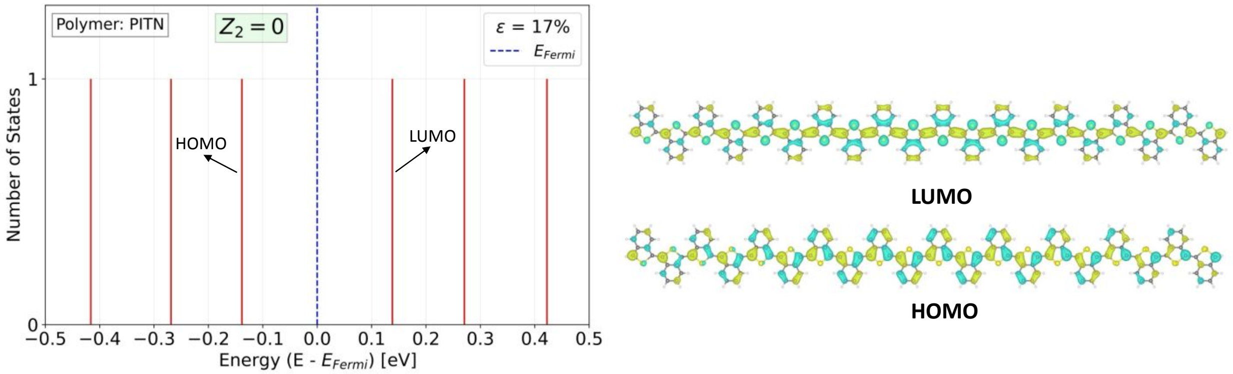

Figure S19: Trivial states of PITN at +17.0% strain values. No edge states are observed in this case. The density of states is symbolic since there are continuous levels below the HOMO and above the LUMO, which are replaced by a finite k-point grid. The HOMO is antibonding (out of phase) between the repeat units while LUMO is bonding (in-phase). Isovalue = 0.00007 a.u.

4. BLA and bandgap

Bond length alternation (BLA) is a purely geometry-based parameter defined by the bond length alternation along a chain of sp^2 carbon atoms according to equation (2) in the main text. Figure S20 shows that in cases where it is a well-defined single alternating chain of carbon atoms all polymers comply with the general trend that BLA>0 describes an aromatic, and BLA<0 a quinonoid structure. However, for polymers with a topology that lacks this well-defined chain of carbon atoms, such as PF and BdiT, the identification of whether the structure is quinonoid or aromatic is ambiguous due to the six-membered benzene rings that has two parallel pathways of conjugation along the carbon backbone. In such cases, the topological Z_2 Zak invariant[1] is still applicable showing the superiority of the later.

The correlation between BLA and the bandgap is indirect with the exception of polyacetylene, as expressed by equation (1). In general, heteroatoms, conjugated rings as in PT, and complex topologies, as in TTD, affect the correlation.[2] Hence, only an overall trend can emerge, as illustrated in Figure S21.

Figure S22 shows for five selected cases how the changes of BLA come about resulting from the changes of the individual CC bonds during expansion/compression of the polymer. All bond distances increase as strain changes from -5 % to +5 % as one would expect based on the overall change of the length of the unit cell along the translation vector, $\vec{l}$. However, BLA can increase or decrease depending on the relative changes of the various r_i bond distances as $\vec{l}$ is increased.

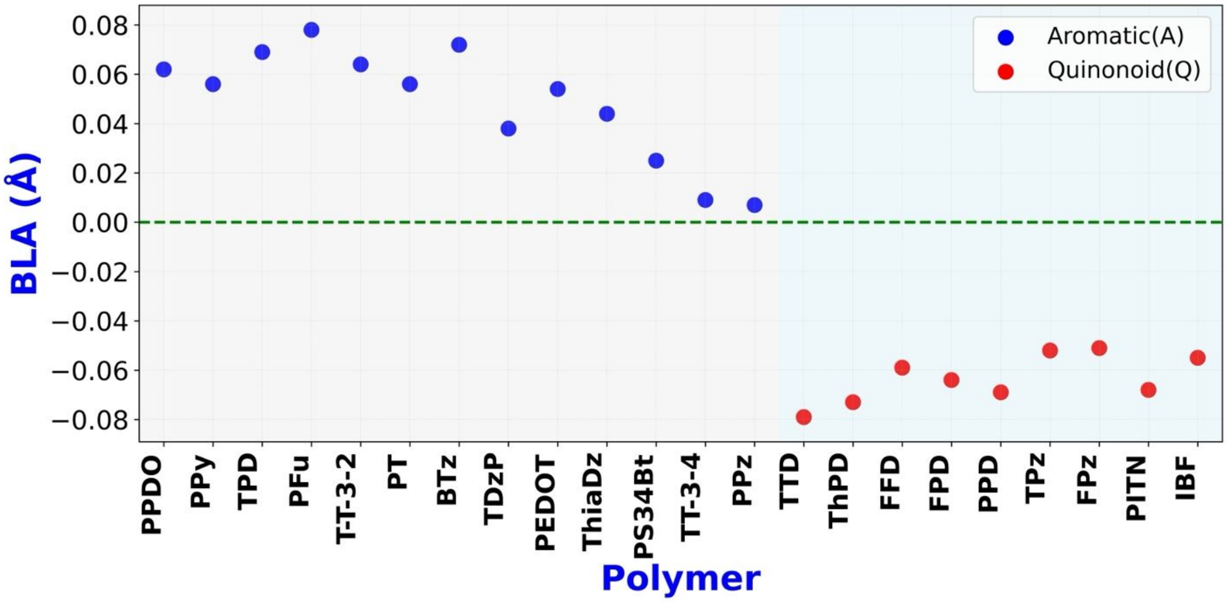

Figure S20. Bond length alternation (BLA) of all polymers at zero strain except PF and BdiT. The order of the systems from left to right is the same as in Figure 6 in the main text.

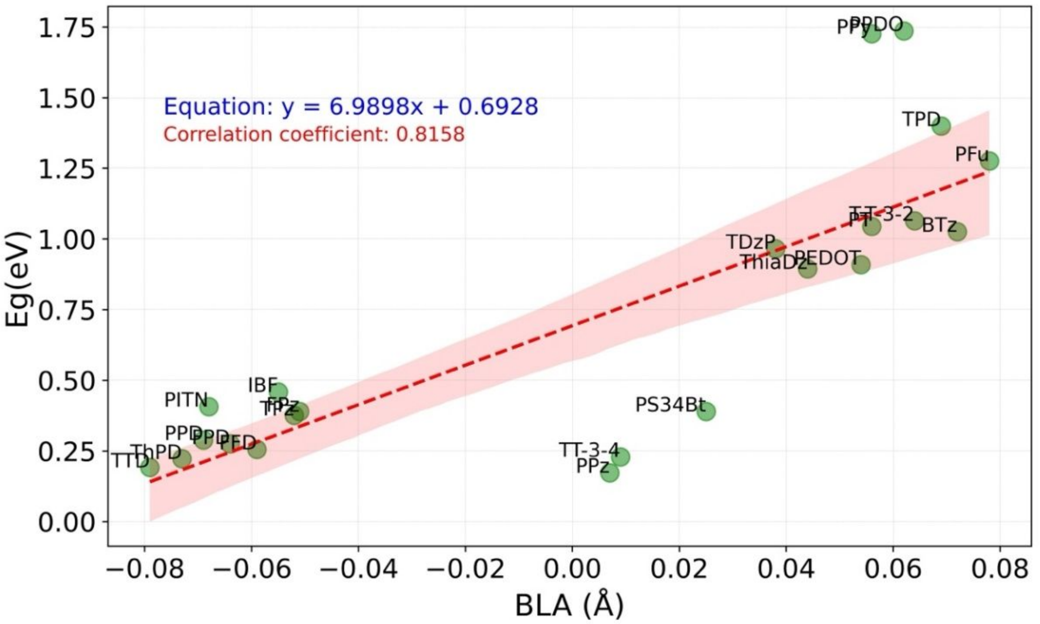

Figure S21. Bandgaps (E_g) of 24 polymers discussed in the main text at zero external strain as a function of bond length alternation (BLA). The pink zone around the regression line is a confidence interval (CI) with a CI value of 0.95.

The bond length alternation (BLA) versus strain is shown for five cases in Figure S22. The overall slopes, and the slopes at zero strain are positive for two and negative for three. For TPz the BLA is negative (quinonoid structure) and the positive slope indicates that tensile strain can reduce this BLA. The gap therefore is also reduced with tensile strain in this case, and the topological transition (level crossing) is achieved. The qualitative description is mirrored for the case of PT, and aromatic system, in the opposite direction: at zero strain BLA>0, so in order to reduce the BLA (and the gap) the polymer needs to be compressed to achieve the gap reduction and the topological transition to the quinonoid phase. PPD, FPD, and TTD are three quinonoid systems out of the five in Figure S22 that behave differently. These three differ in two ways. First, the absolute value of the slope is much smaller, indicating that the BLA will change less with strain, indicating a likely smaller coupling to the energy levels and the gap. Second, the sign of the slope is negative, the opposite of that of TPz. As a consequence, tensile strain increases the gap (compressive reduces it) as seen in the signs of the coupling, κ, shown in Figures 7 and S23.

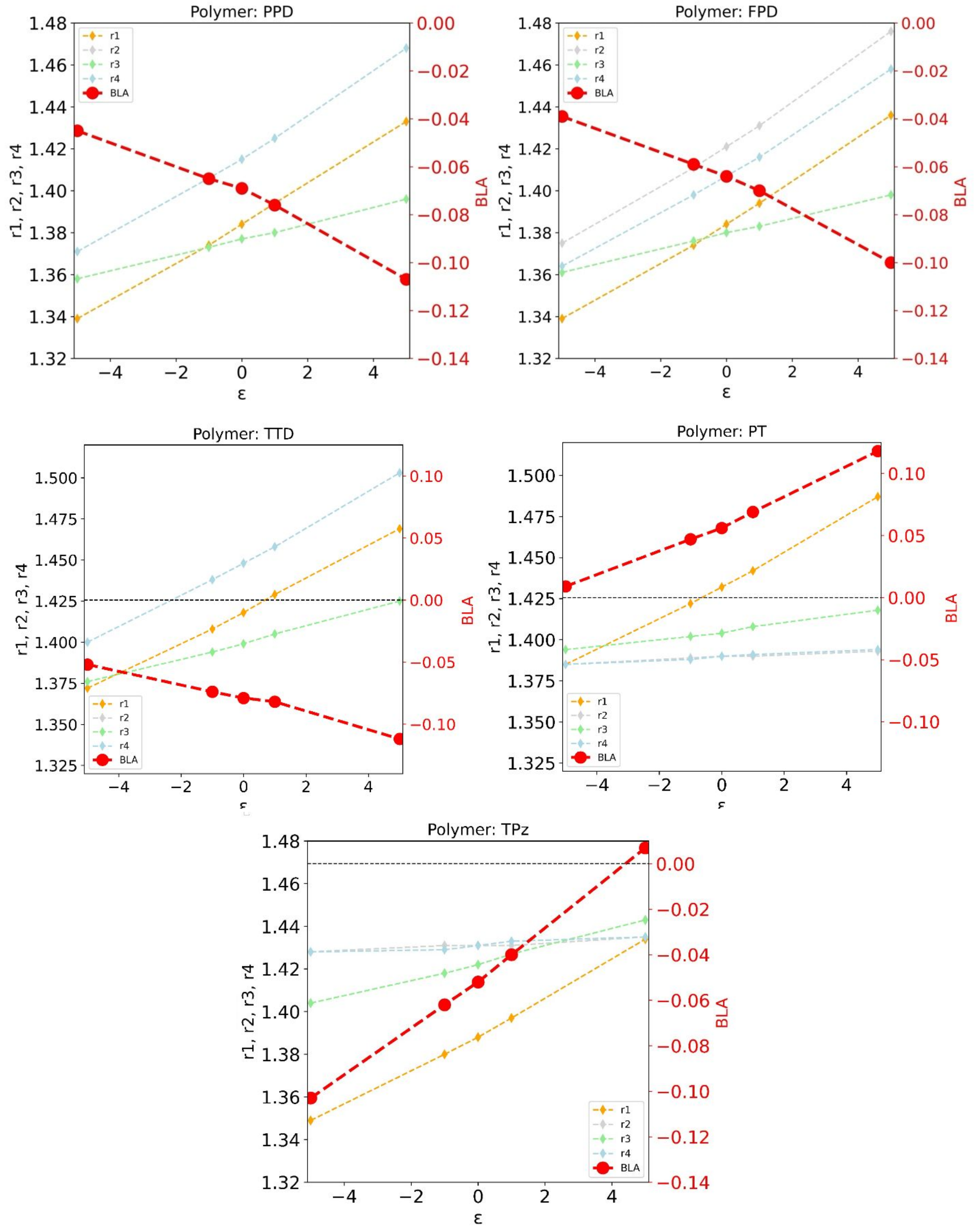

Figure S22. Changes of BLA in PPD, FPD, TTD, PT, and TPz polymer as the longitudinal strain varies from -5% to +5%. The distances r_1, r_2, r_3, and r_4 are used to calculate BLA by eq. (2). For PPD $r_2 = r_4$ due to symmetry.

5. Bandgap changes and figure of merit at $\varepsilon = \pm 5$ %

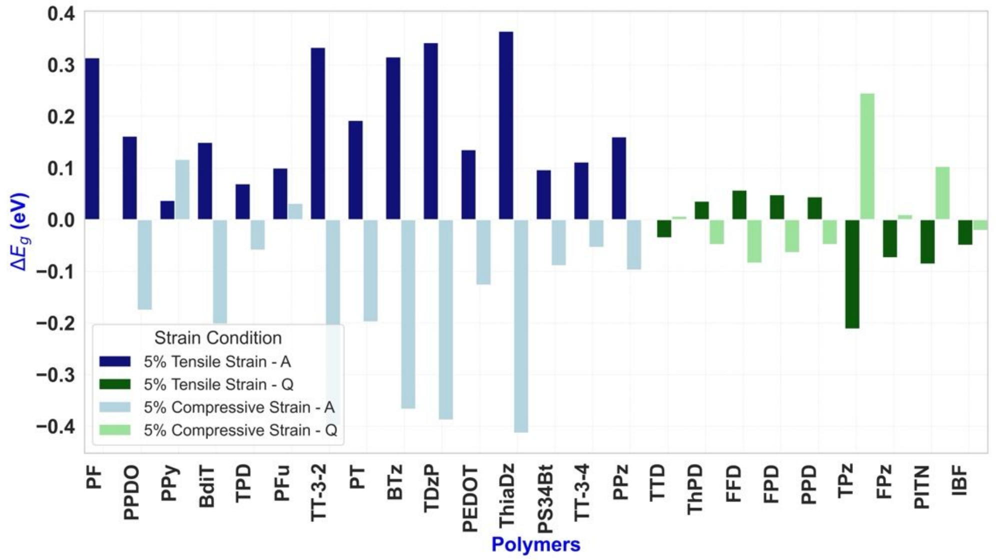

Figure S23. Change of bandgap at $\varepsilon = \pm 5$ %. $\Delta E_g = E_g(\varepsilon) - E_g(\varepsilon = 0)$. All polymers discussed in order as listed in Figure 6. Note that structure of PF polymer with 5% compressive strain is not listed as the structure becomes non-planar in the geometry optimization.

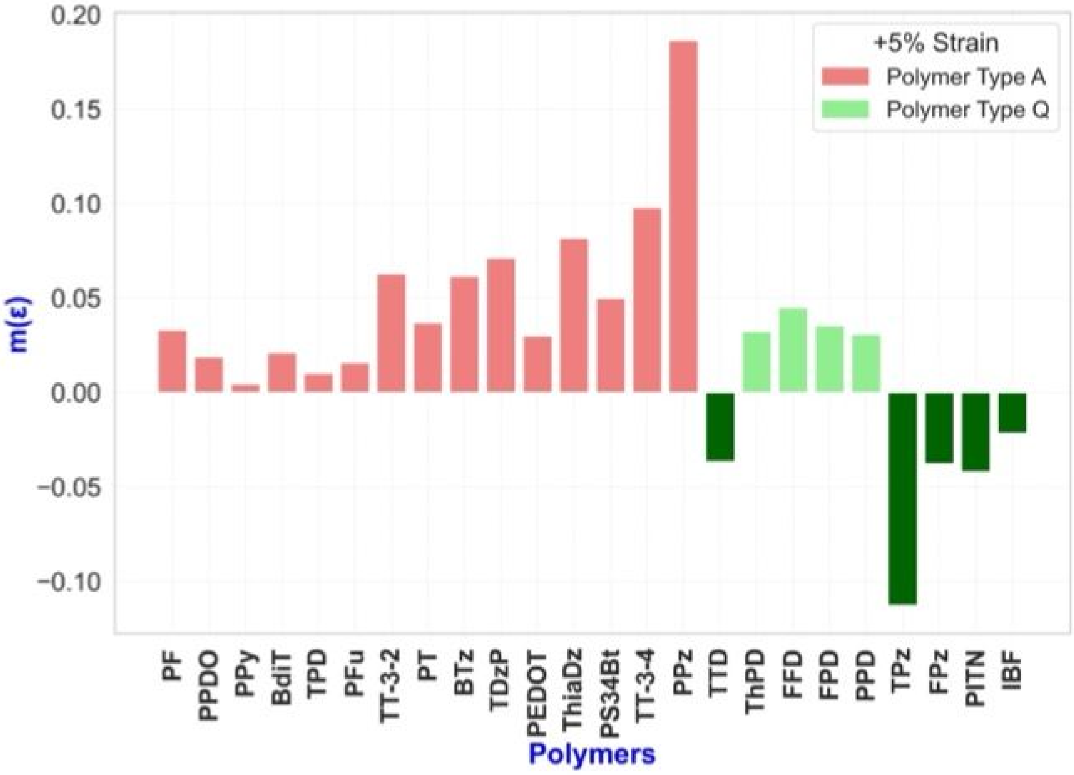

Figure S24. Figure of merit, m, as defined by eq. (3) for all polymers listed in Figure 1 at ε=+5 % strain. The polymers are listed in the same order as in Figure 5 in the main text.

6. The nature of level crossing upon strain

The change of the bandgap as a function of strain is an effect that relies on the level crossing of the HOMO and LUMO frontier orbitals upon strain. This effect depends strongly on the electronic structure of the individual polymer. This is the reason why the $\kappa(\varepsilon)$ couplings and the m (ε) figure of merit values vary dramatically from polymer to polymer, as is clear from their values displayed in Figures 7 and 8. Figure S25 displays extreme cases of strong (TPz) vs. weak (PFu, PPy, and IBF) dependencies of the gap as a function of strain.

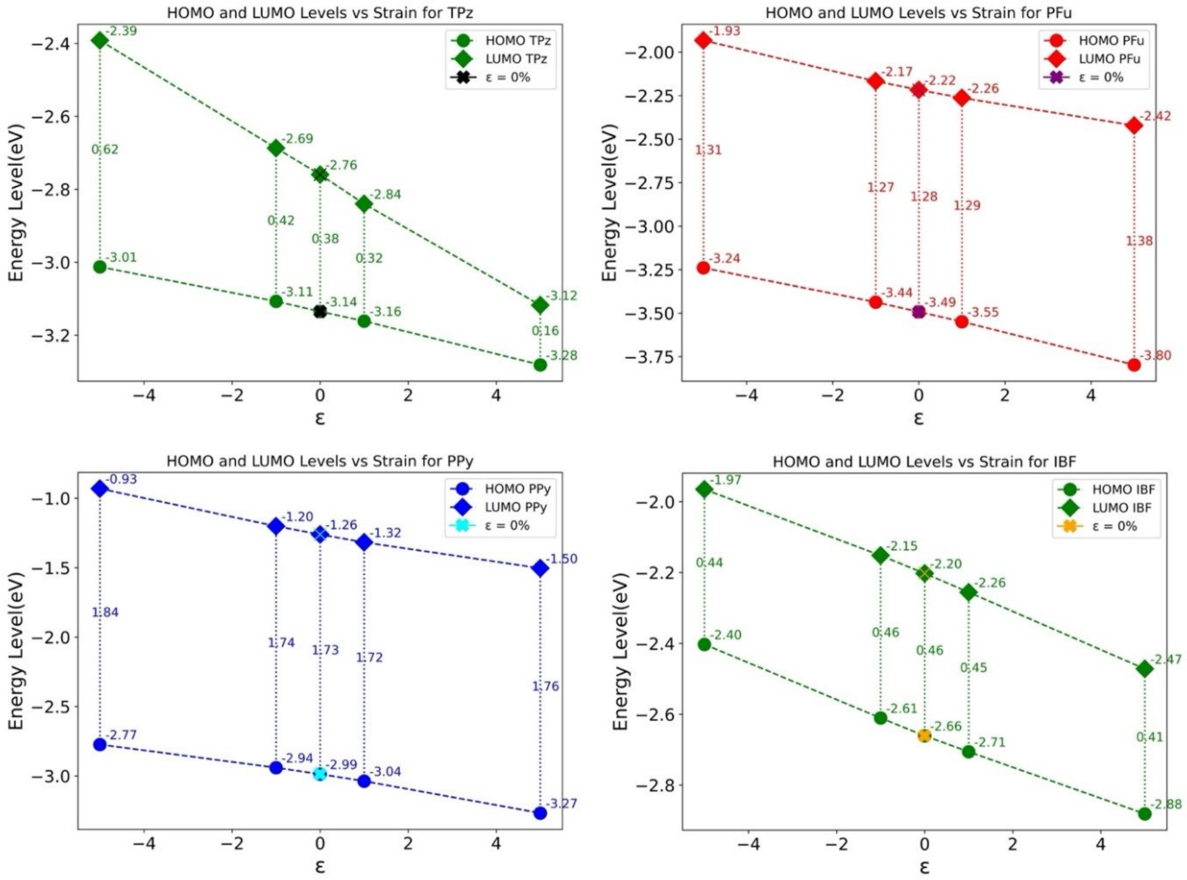

Figure S25. Selected HOMO and LUMO as a function of strain (ε). Note the strong dependency of the gap (TPz) vs. the very weak (PFu, PPy, and IBF) dependency on strain.

7. Young's modulus for the considered polymers

The Young's modulus (Y) is computed from the second derivative of the total energy of the system with respect to strain across the polymer's cross-section:[3]

$$Y = \frac{1}{A}\left(\frac{d^2E}{d\varepsilon^2}\right) \quad \text{(S1)}$$

where A is the cross-sectional area of the polymer, and E is the total energy per unit cell. The cross-sectional area is considered appropriate in this context. The calculated Young's modulus values, assuming A=40 Å^2, for six representative π-conjugated polymers are provided below.

These values are consistent with each other and in comparison with those of polyacetylene (PA), poly(methylene oxide) (PMO), polyethylene (PE), and other organic polymers, indicating the overall consistency of the computational methods used.[4-7]

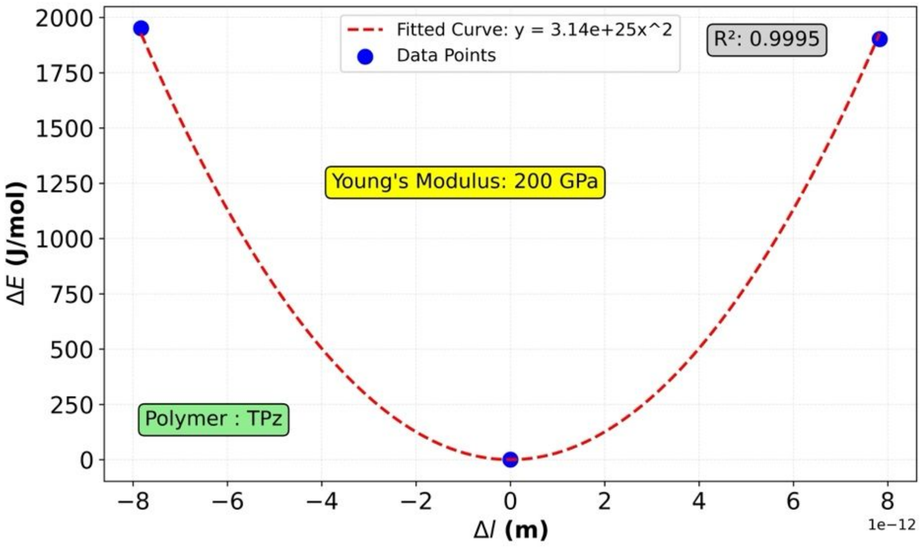

Figure S26: Young's modulus (Y) for the TPz polymer, calculated from the energy versus strain plot fitted with a second-degree polynomial.

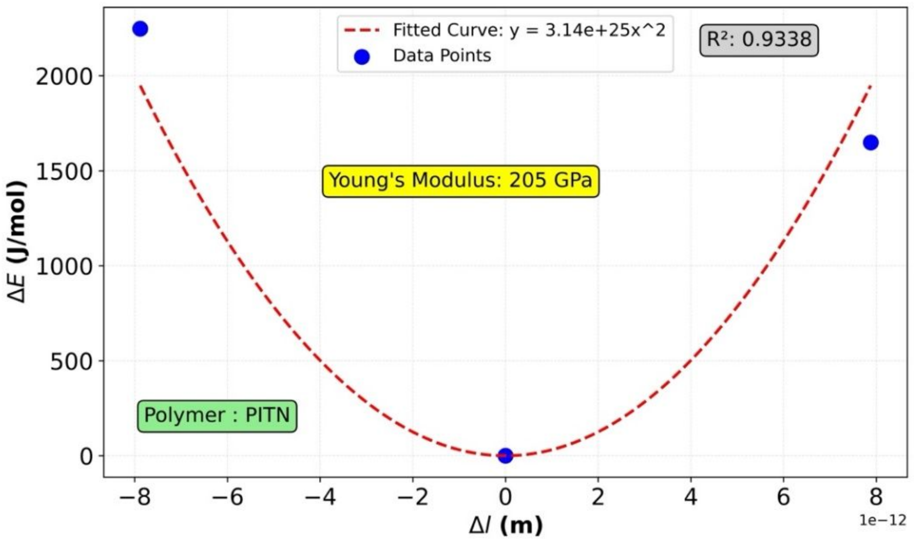

Figure S27: Young's modulus (Y) for the PITN polymer, calculated from the energy versus strain plot fitted with a second-degree polynomial.

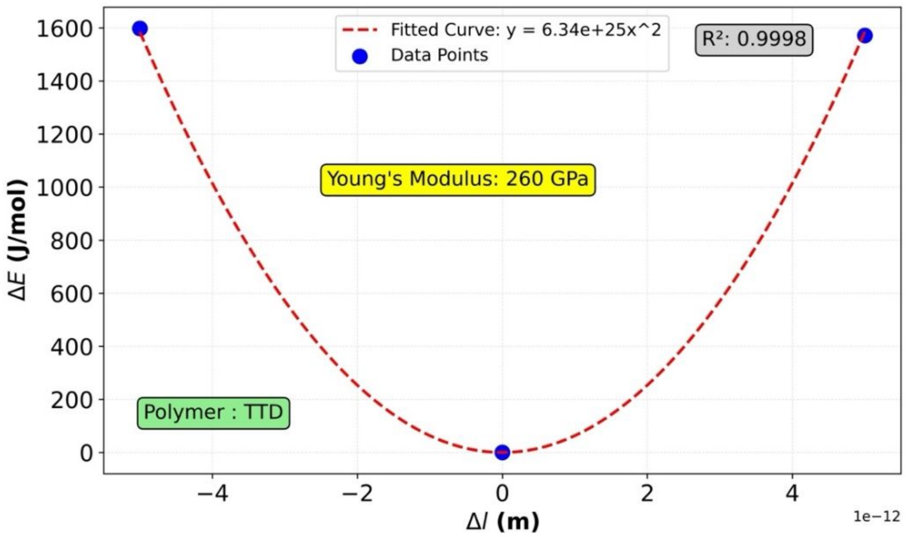

Figure S28: Young's modulus (Y) for the TTD polymer, calculated from the energy versus strain plot fitted with a second-degree polynomial.

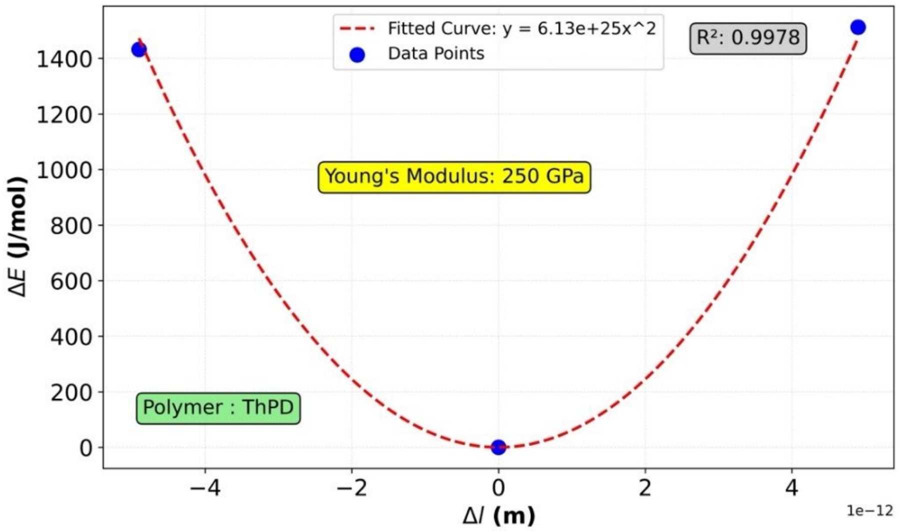

Figure S29: Young's modulus (Y) for the ThPD polymer, calculated from the energy versus strain plot fitted with a second-degree polynomial.

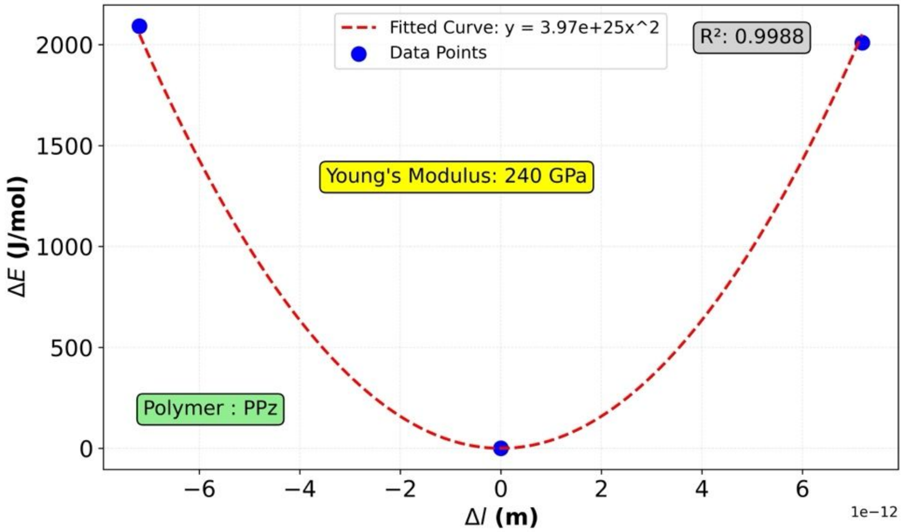

Figure S30: Young's modulus (Y) for the PPz polymer, calculated from the energy versus strain plot fitted with a second-degree polynomial.

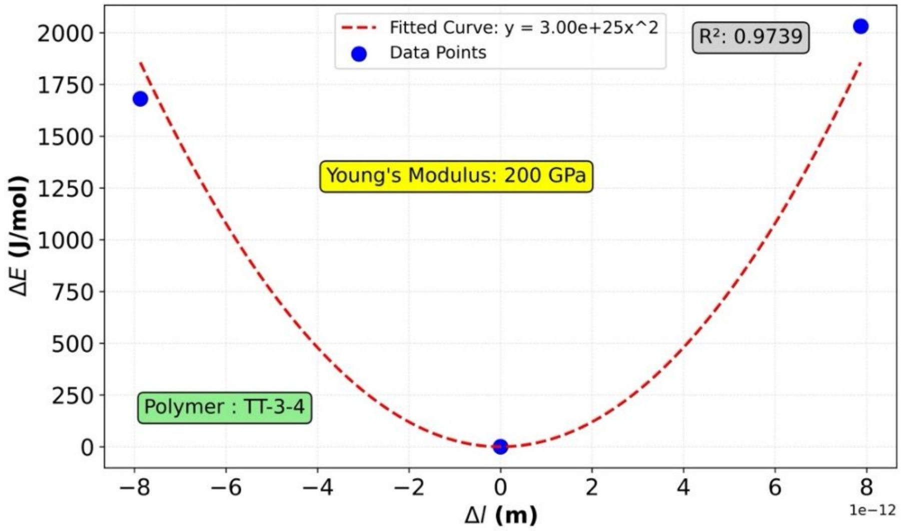

Figure S31: Young's modulus (Y) for the TT-3-4 polymer, calculated from the energy versus strain plot fitted with a second-degree polynomial.

8. Correlation between PBE and PBE0

The pure PBE DFT functional often underestimates the bandgap, so we also calculated the bandgap (E_g) for all polymers at 0% strain using the PBE0 functional.[8] For these PBE0 calculations, we switched from ultrasoft (US) pseudopotentials[9] to norm-conserving (NC)

pseudopotentials[10] and used an energy cutoff of 90 Ry due to the high computational cost of the former. This setup made the calculations faster and feasible in terms of time. The PBE0 functional includes 25% of the exact exchange (EXX)[11] with higher computational costs. Therefore, we primarily relied on PBE, which has shown considerable efficacy in periodic computations. The bandgap (E_g) computed using PBE and PBE0 shows an excellent correlation, as illustrated in Figure 27 for our 24 polymers.

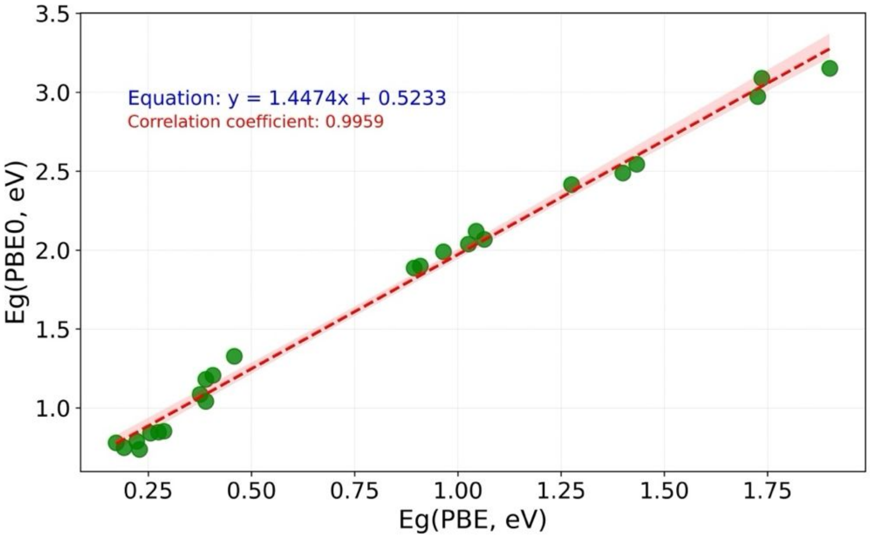

Figure S32: The correlation between the bandgap (E_g) obtained from PBE and PBE0 (Exact Exchange = 25%). PBE0 computations were done at the PBE optimized geometries at zero strain.

9. Introduction of torsion along the polymer chain.

Recently it has been indicated that even minor changes in the torsion angle of the aromatic rings can lead to shifts in the topological state of the polymer.[12] We show that in our cases this effect does not occur, because our systems are at their energy minima. The figures below illustrate this circumstance.

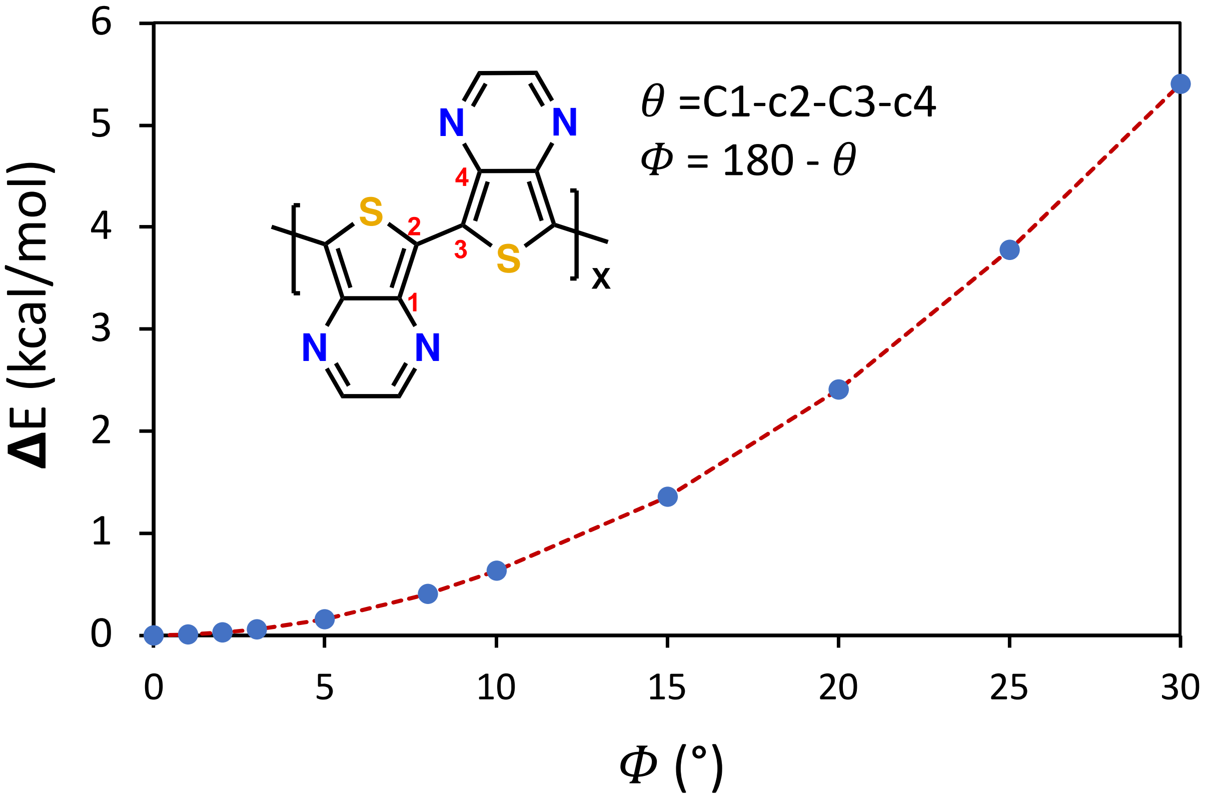

Figure S33: Potential Energy Surface (PES) of the TPz polymer as a function of the dihedral angle, Φ.

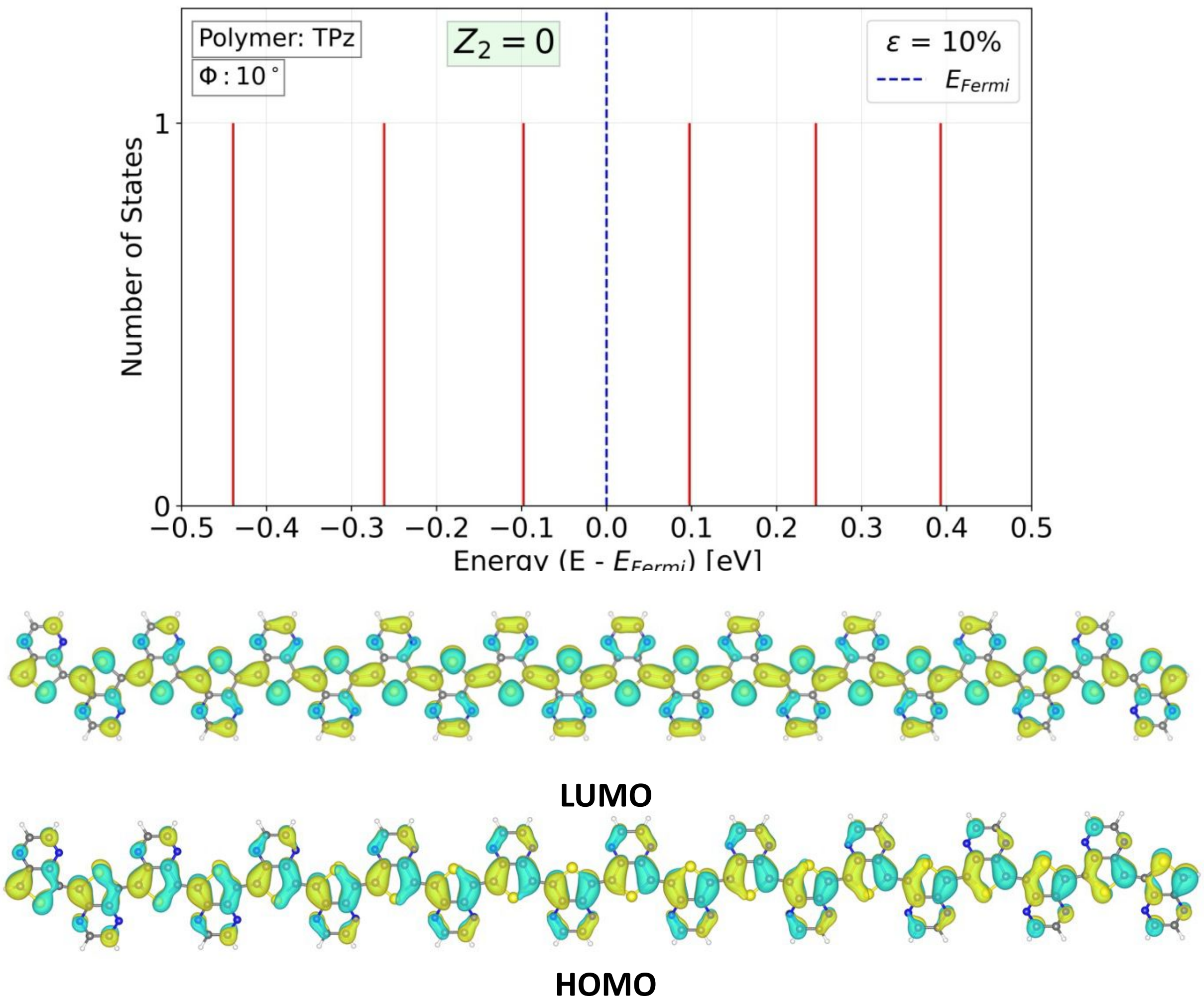

Figure S34: Trivial (Z_2=0) phase polymer TPz (at ε=10% and Φ=10°) which is aromatic. No edge state is observed for the polymer. The number of states is symbolic, with continuous levels below the HOMO and above the LUMO not being indicated here.

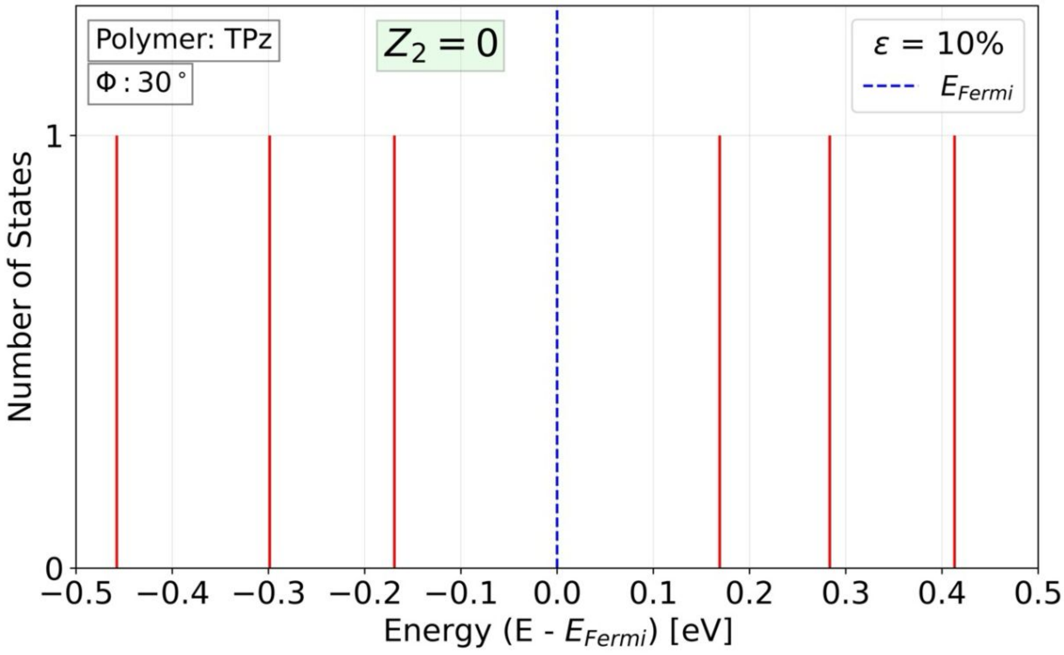

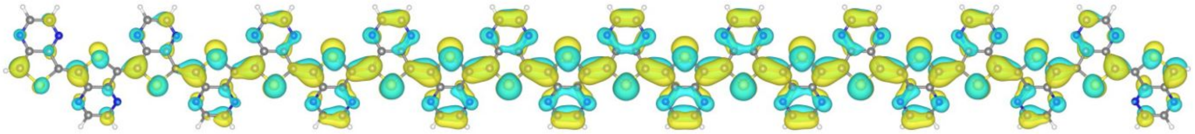

LUMO

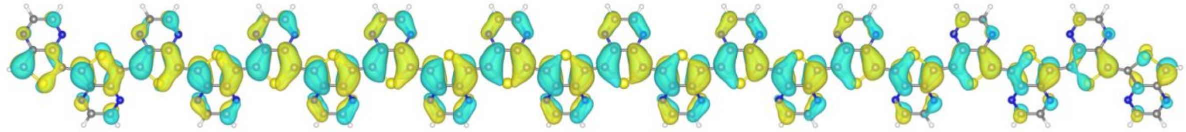

HOMO

Figure S35: Trivial (Z_2=0) phase polymer TPz (at ε=10% and Φ=30°) which is aromatic. No edge state is observed for the polymer. The number of states is symbolic, with continuous levels below the HOMO and above the LUMO are not being indicated here.

10. Using the figure of merit, $m(\varepsilon)$, to estimate the critical strain value

Assuming that $E_g(\varepsilon)$is approximately linear as a function of ε up to the point where $E_g(\varepsilon) = 0$, we can make the following estimate:

$$E_g(\varepsilon) = E_g(\varepsilon = 0) - s\varepsilon \quad \text{(S2)}$$

where s is the slope. It can be approximated by a finite difference at 1% strain:

$s = \frac{\Delta E_g}{\varepsilon} \approx \kappa(1\ \%)$ (S3)

The gap vanishes therefore approximately at

$E_g(\varepsilon) = E_g(\varepsilon = 0) - \kappa\varepsilon = 0$
(S4)

This occurs when $E_g(\varepsilon = 0) \approx \kappa(1\%)\varepsilon_T$, with subscript T for the transition.

Approximately at

$\varepsilon_T = \frac{E_g(\varepsilon=0)}{\kappa(1\%)} = 1/m(1\%)$ (S5)

For example, for TPz, the transition is estimated to occur at 7% elongation. In fact, the actual computation gives the more accurate value of 9.2% as shown in Figure 3.

11. References

(1) Cao, T.; Zhao, F.; Louie, S. G. Topological phases in graphene nanoribbons: junction states, spin centers, and quantum spin chains. *Phys. Rev. Lett.* **2017**, *119* (7), 076401.
(2) Kertesz, M.; Choi, C. H.; Yang, S. Conjugated polymers and aromaticity. *Chem. Rev.* **2005**, *105* (10), 3448-3481.
(3) Hong, S. Y.; Kertesz, M. Dependence of Young's modulus of trans-polyacetylene upon charge transfer. *Phys. Rev. Lett.* **1990**, *64* (25), 3031.
(4) Kurita, T.; Fukuda, Y.; Takahashi, M.; Sasanuma, Y. Crystalline moduli of polymers, evaluated from density functional theory calculations under periodic boundary conditions. *ACS omega* **2018**, *3* (5), 4824-4835.
(5) Li, P.; Hu, L.; McGaughey, A. J.; Shen, S. Crystalline polyethylene nanofibers with the theoretical limit of Young's modulus. *Adv. Mater.* **2014**, *26* (7), 1065-1070.
(6) Chudinov, V. S.; Shardakov, I. N.; Ivanov, Y. N.; Morozov, I. A.; Belyaev, A. Y. Elastic Modulus of a Carbonized Layer on Polyurethane Treated by Ion-Plasma. *Polymers* **2023**, *15* (6), 1442.
(7) Akagi, K.; Sakamaki, K.; Shirakawa, H.; Kyotani, H. Polyacetylene films prepared by intrinsic non-solvent polymerization method-mechanical properties and electrical conductivities. *Synth. Met.* **1995**, *69* (1-3), 29-30.
(8) Perdew, J. P.; Burke, K.; Ernzerhof, M. Generalized gradient approximation made simple. *Phys. Rev. Lett.* **1996**, *77* (18), 3865.
(9) Vanderbilt, D. Soft self-consistent pseudopotentials in a generalized eigenvalue formalism. *Phys. Rev. B* **1990**, *41* (11), 7892.
(10) Hamann, D. Optimized norm-conserving Vanderbilt pseudopotentials. *Phys. Rev. B* **2013**, *88* (8), 085117.
(11) Barnes, T. A.; Kurth, T.; Carrier, P.; Wichmann, N.; Prendergast, D.; Kent, P. R.; Deslippe, J. Improved treatment of exact exchange in Quantum ESPRESSO. *Comput. Phys. Commun.* **2017**, *214*, 52-58.

(12) Alcón, I.; Canonico, L. M.; Papior, N.; Garcia, J. H.; Cummings, A. W.; Tremblay, J. C.; Pruneda, M.; Brandbyge, M.; Paulus, B.; Roche, S. Twisting Between Topological Phases in 1D Conjugated Polymers via a Multiradical Transition State. *Adv. Funct. Mater.* **2024**, 2409174.
